# Epidural Adhesiolysis and Dorsal Root Ganglion Pulsed Radiofrequency Through a Single Multifunctional Catheter: A Narrative Review and Illustrated Technical Report

**DOI:** 10.3390/jcm15208094

**Published:** 2026-10-19

**Authors:** Giuliano Lo Bianco, Miles Day, Douglas P. Beall, Sudhir Diwan, Alaa Abd-Elsayed, Matteo Luigi Giuseppe Leoni, Felice Occhigrossi, Marco Mercieri, Marco Cascella, Francesco Paolo D’Angelo

**Affiliations:** 1 Unit of Anesthesia, Analgesia and Percutaneous Surgery, Fondazione Istituto G. Giglio, Cefalù, 90015 Palermo, Italy; francescods.dangelo@gmail.com; 2 Department of Anesthesiology and Pain Medicine, Texas Tech University Health Sciences Center, Lubbock, TX 79430, USA; miles.day@ttuhsc.edu; 3 Comprehensive Specialty Care, Oklahoma City, OK 73112, USA; db@clinrad.org; 4 Advanced Spine on Park Avenue, New York, NY 10022, USA; sudhir.diwan63@gmail.com; 5 Department of Anesthesiology, University of Wisconsin School of Medicine and Public Health, Madison, WI 53726, USA; abdelsayed@wisc.edu; 6 Department of Medical and Surgical Sciences and Translational Medicine, Sapienza University of Rome, 00185 Rome, Italy; matteolg.leoni@gmail.com (M.L.G.L.); marco.mercieri@uniroma1.it (M.M.); 7 Pain Therapy Unit, Azienda Ospedaliera Sandro Pertini, 00157 Rome, Italy; focchigrossi@hsangiovanni.roma.it; 8 Department of Medicine, Surgery and Dentistry, University of Salerno, 84081 Baronissi, Italy; mcascella@unisa.it

**Keywords:** epidural adhesiolysis, epidurolysis, hyaluronidase, pulsed radiofrequency, dorsal root ganglion, epidurography, persistent spinal pain syndrome, interventional pain medicine

## Abstract

Percutaneous epidural adhesiolysis and pulsed radiofrequency of the dorsal root ganglion (DRG-PRF) target two distinct components of chronic lumbosacral radicular pain: the restricted access of injected drugs to a nerve root shielded by epidural fibrosis, and the sensitization of the ganglion itself. A multifunctional steerable catheter carrying a bipolar electrode at its tip makes it technically feasible to perform both at the same anatomical target within a single procedure. This narrative review and illustrated technical report has three aims: to summarize the mechanistic rationale for each component (mechanical adhesiolysis, enzymatic lysis with recombinant human hyaluronidase, DRG-PRF, and the co-administered local anesthetic and corticosteroid); to describe the combined procedure step by step—landmarks, needle angles, radiofrequency parameters, injectate composition, safety checkpoints and the epidurographic endpoint—with original schematics; and to set out critically what the evidence does and does not establish. A structured but non-systematic search of MEDLINE and Embase identified a substantial, methodologically contested literature on adhesiolysis, a smaller one on hyaluronidase, and a single randomized trial of 41 patients directly comparing adhesiolysis with and without DRG-PRF. Mechanistic plausibility and technical feasibility are therefore well supported, whereas evidence of clinical benefit for the combined protocol is limited. The suggested place of the technique in the treatment pathway—as an interventional option in selected patients before surgical escalation, or when revision surgery is being considered—is a clinical proposal based on anatomical reasoning and single-center experience, not on comparative data. Comparative studies are needed to define its role.

## 1. Introduction

Low back pain is the leading cause of years lived with disability worldwide [1], and most interventional treatments for its radicular form assume that a drug delivered into the epidural space will reach the root that hurts. In some patients, that assumption is wrong: fibrous tissue laid down after surgery, bleeding, or prolonged inflammation compartmentalizes the epidural space [2,3,4], and an injectate delivered from a distance flows around the obstruction rather than through it. The clinical signature is a patient whose imaging shows a plausible target, whose pain follows a dermatomal distribution, and who obtains little relief from a technically correct epidural injection [3]. Percutaneous epidural adhesiolysis was introduced to address this problem of access—by separating the scar mechanically, by softening it enzymatically, or by both—so that an injected drug can reach the root.

Restoring access does not, however, reverse the changes that a chronically irritated root has undergone. Sustained nociceptive input induces maladaptive plasticity in the dorsal root ganglion and dorsal horn, and once these changes are established, a transient anti-inflammatory effect at the root may leave them untouched [5,6]. Pulsed radiofrequency of the dorsal root ganglion offers a non-lesional way of modulating the sensitized ganglion [7]. Combining it with adhesiolysis—one addressing the environment of the root, the other the neuron—is mechanistically coherent and matches the wider case for choosing an intervention by the mechanism that sustains the pain rather than by the anatomical site alone [8]; but until recently it required separate devices and separate procedures, and the established neuromodulation techniques that target the ganglion remain unevenly accessible [9]. A multifunctional steerable catheter that carries a bipolar electrode at its tip removes the first constraint. Two cautions frame what follows: mechanistic complementarity does not by itself imply additive or synergistic clinical benefit, and the direct evidence for the combined approach consists of a single randomized trial of 41 patients [10].

The replacement of “failed back surgery syndrome” by persistent spinal pain syndrome (PSPS), with its type 1 (no previous surgery) and type 2 (after surgery) [11,12], has a technical as well as a nosological dimension, because the two types tend to differ in the state of the epidural space and therefore in which component of a combined procedure is likely to be doing the work. How far that distinction can be used to guide procedural decisions, and with what evidence, is discussed in Section 4.5.

This article has two purposes. The first is to bring together the mechanistic rationale for each component of the combined procedure, evidence that is otherwise dispersed across four separate techniques, and to place it against what the clinical literature does and does not establish. The second is to describe the procedure as it is performed at our center, with the landmarks, angles, parameters, sequence, and checkpoints needed for another operator to reproduce it. The article is a narrative review with an illustrated technical report: it is not a systematic review, and it does not report clinical outcomes, which for our consecutive cohort are reported in a separate manuscript currently under review [13] and are cited here as unpublished data, only with their limitations stated.

## 2. Methods

### 2.1. Design

This is a narrative review combined with a technical report. The literature was identified through a structured search, described below, but the review is not systematic: no protocol was registered, study selection was performed by a single reviewer (G.L.B.) and was not duplicated, and no formal risk-of-bias assessment was performed. The evidence summarized in Section 3 and Section 4 should therefore be read as a structured orientation to the literature rather than as a synthesis, and the PRISMA-style diagram in Section 3 documents the provenance of the cited references rather than a systematic screening process. The technical description reflects the institutional protocol in use at the Fondazione Istituto G. Giglio, Cefalù, Italy; where a parameter or a step derives from local practice rather than from comparative evidence, this is stated.

### 2.2. Sources and Search Strategy

**Figure 1:**
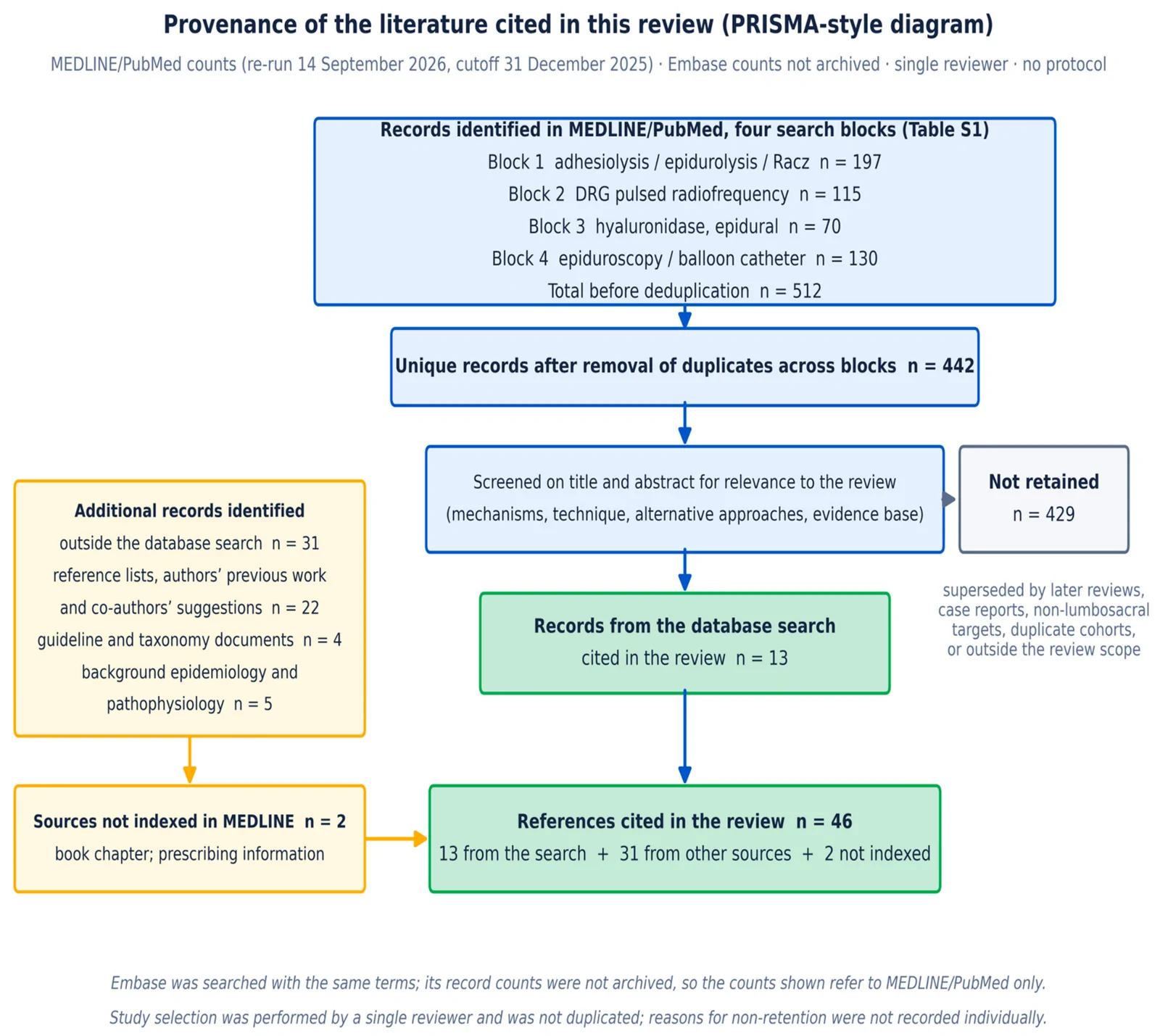
Provenance of the literature cited in this article (PRISMA-style diagram). Counts refer to the MEDLINE/PubMed search re-run on 14 September 2026 with a cutoff of 31 December 2025; Embase counts were not archived. The diagram documents where the cited references came from; it does not represent a systematic screening process.

MEDLINE (via PubMed) and Embase were searched from inception to 31 December 2025. These two databases were chosen because they are the largest and have complementary coverage—MEDLINE for the core clinical and neuroscience journals, Embase for European journals, device-related literature, and conference abstracts—which matters for a technique developed largely in Europe and supported by a sparse, device-specific evidence base; the Cochrane trial register draws on both and was not searched separately. Four concept blocks combined Medical Subject Headings with free-text terms for (i) epidural adhesiolysis, epidurolysis, and the Racz technique, (ii) dorsal root ganglion pulsed radiofrequency, (iii) epidural hyaluronidase, and (iv) epiduroscopy and balloon catheters; the complete strings and MeSH terms are given in Table S1 (Supplementary Material S1). The MEDLINE search was re-run on 14 September 2026, with the same cutoff of 31 December 2025, to generate the record counts reported in Section 3. The Embase record counts were not archived at the time of the original search and could not be reconstructed; the counts in Figure 1 therefore refer to MEDLINE/PubMed only. Reference lists of the retrieved articles, the authors’ own files, and suggestions from co-authors were screened by hand; the four references published in 2026 [7,8,14,15] were identified in this way, outside the database search.

### 2.3. Selection of Publications

Publications were considered if they were in English, concerned adults with lumbosacral radicular or post-surgical spinal pain, and addressed one of the four components of the combined procedure, the combination itself, or one of the alternative adhesiolysis techniques. Within this scope, randomized trials, systematic reviews, and the guideline documents of professional societies were retained whenever found; original observational studies were retained when they addressed predictors of response, safety, or an alternative technique (balloon catheter, epiduroscopy) for which no higher-level evidence exists. Contradictory and negative evidence was actively sought: systematic reviews and guidelines that do not recommend adhesiolysis, and the correspondence they provoked, were retrieved and are presented alongside the favorable literature. Where a systematic review covered a body of primary studies, the review is cited rather than its constituent trials, except for the randomized trials that are discussed individually in Section 4. Records were excluded when they concerned non-lumbosacral targets, duplicate reports of the same cohort, case reports without a safety signal, or were superseded by a later review from the same group. Because selection was performed by one reviewer without a pre-specified protocol, these criteria describe the reasoning applied rather than a reproducible algorithm, and the limitations that follow from this are acknowledged in Section 4.5.

### 2.4. Use of Artificial Intelligence

During the preparation of this manuscript, the authors used a large language model (Claude, Anthropic; version Claude Fable 5.1, 2026, accessed through the Claude desktop application) for language editing of author-written text, for generating the code that draws the original schematic figures from anatomical content, parameters, and concepts specified by the authors, and for formatting the reference list, including checking each reference against PubMed. The model was not used to generate scientific content, clinical recommendations, or conclusions, and no fluoroscopic or intra-operative image was created or altered by artificial intelligence. The authors reviewed and edited all output and take full responsibility for the content of this publication.

## 3. Overview of the Evidence Identified

The four MEDLINE/PubMed search blocks returned 512 records (197 for adhesiolysis and the Racz technique, 115 for dorsal root ganglion pulsed radiofrequency, 70 for epidural hyaluronidase and 130 for epiduroscopy and balloon catheters), of which 442 were unique after removal of duplicates across blocks (Figure 1). Of these, 429 were not retained for the reasons given in Section 2.3, and 13 are cited in this article. The article cites 46 references in total: 13 retrieved by the database search, 31 identified through other sources (reference lists, the authors’ previous work, co-authors’ suggestions, and the guideline, taxonomy and background literature on epidemiology and pathophysiology), and two that are not indexed in MEDLINE (a book chapter and a manufacturer’s prescribing information). The figure of 46 is therefore the number of references cited, not the number of studies selected through the database search.

The principal clinical studies on which the evidence base rests are listed in Table 1. They fall into three groups. For percutaneous adhesiolysis, there are two randomized trials—the two-year comparison with caudal injection by Manchikanti and colleagues [16] and the small trial of hyaluronidase by Yousef and colleagues [17]—together with several systematic reviews that reach opposite conclusions [18,19], the commentary that one of them provoked [20], and two retrospective cohorts that describe predictors of response [21,22]. For the balloon-inflatable catheter, there is a case series [23]. For the combination of adhesiolysis with dorsal root ganglion pulsed radiofrequency, there is one randomized trial of 41 patients followed for six months [10]. Safety has been addressed in a dedicated retrospective series of 250 patients [24] and in a two-center series of 543 patients [25]. The studies are described here; their interpretation, and the disagreements between them, are discussed in Section 4.

## 4. Discussion and Literature Review

### 4.1. Evidence on the Individual Components

#### 4.1.1. Percutaneous Adhesiolysis

Adhesiolysis is the best studied of the three components, but its evidence base is also the most contested. Systematic reviews from the interventional pain literature covering post-surgical lumbar pain and spinal stenosis converge on the same pattern: a benefit that is largest in the opening three to six months and then decays, without disappearing, across the remainder of the first year. They conclude that the procedure is effective and carries a low complication rate when performed by trained operators [18,20]. An earlier review by Racz and colleagues reached similar conclusions on safety and efficacy while emphasizing the heterogeneity of protocols [26]. In the randomized trial of Manchikanti and colleagues, 120 patients with post-lumbar-surgery syndrome were assigned to adhesiolysis or to a caudal epidural injection; at two years, 82% of the adhesiolysis group had achieved at least 50% relief of pain together with functional improvement, against 5% of controls, at the cost of a mean of 6.4 procedures per patient over the period [16].

Two observational studies are informative about who benefits. Kose and Akkaya studied 193 patients with chronic lumbar radicular pain resistant to epidural steroid injection and found that 56.2% achieved a 50% reduction in pain at twelve months, identifying absence of depression, no previous lumbar surgery, mild rather than severe foraminal stenosis, and absence of spondylolisthesis as predictors of success [21]. Lee and Lee reviewed 65 patients with lumbosacral spinal stenosis refractory to transforaminal injection and found that 54% still had at least 50% relief of back pain at three months, with previous lumbar surgery, spondylolisthesis, and foraminal stenosis again marking those who did not respond [22]. The consistent message is that the technique works best where the obstruction is moderate and the anatomy has not been repeatedly altered.

The evidence is reflected in the guidelines of the American Society of Interventional Pain Physicians, which grade percutaneous adhesiolysis as level I with strong recommendation in post-surgical persistent spinal pain and level II with moderate-to-strong recommendation in central canal stenosis and in disc herniation, radiculitis or severe degenerative disc disease [27].

That assessment is not shared across specialties. A systematic review conducted for a health-technology assessment agency, which searched seven databases through 2017, found no randomized trial of efficacy or cost-effectiveness, judged the two randomized trials of effectiveness to have serious methodological flaws, and concluded that the evidence on the effectiveness and safety of adhesiolysis in failed back surgery syndrome was insufficient [19]; the reply from the interventional community argued that the trials had been misclassified and maintained the opposite conclusion [20]. The American Pain Society’s 2009 guideline on interventional therapies for low back pain likewise found the evidence insufficient to recommend adhesiolysis [28], whereas the guideline of the American Society of Interventional Pain Physicians grades it as level I with strong recommendation in post-surgical persistent spinal pain [27]. The reader should therefore be aware that the recommendations summarized above come from the specialty that performs the procedure, that the most favorable trial [16] reports a between-group difference at two years (82% versus 5%) far larger than is usual for any intervention in chronic spinal pain, and that the question of efficacy against a sham or an equivalent control has not been settled.

#### 4.1.2. Hyaluronidase as an Adjunct

The evidence specific to hyaluronidase is thinner and harder to isolate, because in most protocols it is given together with corticosteroid, local anesthetic and, classically, hypertonic saline, so its independent contribution is rarely estimable. Yousef and colleagues randomized 38 patients with failed back surgery syndrome to caudal steroid and hypertonic saline with or without hyaluronidase. Short-term relief was obtained in both arms, but relief that persisted to the end of follow-up was seen only in the hyaluronidase arm, together with improvement in lumbar range of motion and a decrease in opioid intake [17]. Reviews of the enzyme in chronic pain practice describe a favorable safety profile and support the pharmacokinetic rationale—increased distribution of the co-administered agents—rather than a direct analgesic effect [29,30].

Two things should be kept distinct. The pharmacological spreading effect of hyaluronidase—a larger volume of distribution for whatever is injected with it—is well established and does not depend on this literature. Evidence that this translates into an incremental clinical benefit in a modern adhesiolysis protocol, and in particular in one that omits hypertonic saline, rests on a single small trial [17] and has not been replicated. The practical implication is that hyaluronidase may be expected to help where diffusion is the limiting factor and to add little where it is not, but this is a rationale rather than a demonstrated effect. This is consistent with the recommendation of a recombinant human preparation over animal-derived formulations, which retains greater activity at physiological pH and avoids the immunogenicity of bovine or ovine enzyme [30,31].

#### 4.1.3. Pulsed Radiofrequency at the Dorsal Root Ganglion

The evidence for DRG-PRF in lumbosacral radicular pain is heterogeneous. Randomized comparisons against transforaminal steroid injections have generally shown similar short-term analgesia with a longer-lasting effect for pulsed radiofrequency, while the effect on function has been less consistently reported [32]. The mechanistic literature is comparatively strong: preclinical work has documented changes in immediate early gene expression, in glial activation, and in cytokine balance, and more recently epigenetic modulation of nociceptive signaling has been proposed as a substrate for the durability of the effect [5,14,33,34,35]. Comparable dose-dependent effects have been reported when the same principle is applied to peripheral targets such as the genicular nerves [36].

### 4.2. Evidence on the Combined Approach

The evidence for the combination must be distinguished from the evidence for its components: a benefit of adhesiolysis, of hyaluronidase, or of pulsed radiofrequency taken separately does not establish a benefit of the specific combined protocol described in this article. The single study that tests the combination directly is that of Vigneri et al., who randomized 41 patients with chronic lumbosacral radicular pain and neuropathic features to electrocatheter-mediated high-voltage pulsed radiofrequency plus adhesiolysis or adhesiolysis alone. The combined arm showed a greater fall in NRS at one month (−3.43 versus −1.75; *p* = 0.031) and at six months (−3.34 versus −0.80; *p* = 0.005), with responder proportions of 57% versus 25% and 48% versus 10% at the same timepoints [10]. It is a small, single-center trial with a six-month horizon and did not include hyaluronidase or second-day administration, but it is the only randomized evidence that adding pulsed radiofrequency to lysis changes the outcome, and it used the same two-cycle 240-s protocol described here. No study has tested the three-component protocol (mechanical lysis, hyaluronidase and DRG-PRF) against any comparator.

The only other data on the combined protocol come from our own consecutive cohort of 155 patients treated between 2021 and 2024. These are unpublished data: the full report has been submitted to a peer-reviewed journal and is under review at the time of writing [13], so the figures below are provisional, are given only in summary form, and their limitations must be stated before presenting the results: the study was retrospective and single-center; the three treatment options were allocated by the operator according to pathology and intra-procedural findings, not at random, so that the groups differed in disease as well as in treatment; there was no untreated or sham control; follow-up assessments were not blinded; and the longitudinal analysis was a complete-case analysis of 121 of 155 treated patients. The data come from the same authors who describe the technique here and should not be read as independent confirmation of it. With those caveats, in that cohort mean pain intensity (numerical rating scale, NRS) fell from 7.31 at baseline to 2.98 at one month and 3.81 at six months, and was 5.92 at 18 months; the Oswestry Disability Index (ODI) fell from 47.9% to 26.9% at one month and 34.1% at six months; the proportion of patients with at least a 50% reduction in NRS was 72% at three months, 64% at six months and 10% at 18 months; pain relief did not differ between the three options, whereas the improvement in disability at six months was greater in the two options that included pulsed radiofrequency (adjusted difference in ODI versus mechanical lysis alone, −8.6 and −7.5 points); and no major adverse event was documented in the 155 patients. What these unpublished figures suggest is that the protocol can be delivered consecutively in routine practice with substantial early improvement that attenuates over 18 months; they cannot show what part of the improvement is attributable to any component, nor how the protocol compares with simpler alternatives.

### 4.3. Anatomical Considerations and Proposed Mechanisms

The four components of the procedure are proposed to act on different elements of the same clinical problem. This section sets out the rationale for each and why the combination is coherent. Throughout, a distinction is maintained between established biological mechanisms—the biology of fibrosis, the pharmacology of hyaluronidase, the physics of the radiofrequency field—and proposed procedural mechanisms, which are inferred from anatomy and from epidurographic observation and have not been demonstrated directly in clinical studies.

#### 4.3.1. Mechanical Adhesiolysis with the Catheter

Epidural fibrosis develops when blood, surgical trauma, extruded disc material, or chronic inflammation provoke a fibroproliferative response in the epidural fat, which is progressively replaced by collagenous scar. The cascade is well characterized: an inflammatory phase in which damaged tissue recruits neutrophils, macrophages and lymphocytes that release tumor necrosis factor alpha (TNF-α), interleukin-1 beta (IL-1β), interleukin-6 (IL-6), prostaglandins and leukotrienes; a proliferative phase in which transforming growth factor beta and platelet-derived growth factor drive fibroblast migration and the synthesis of collagen, fibronectin and proteoglycans; wound contraction by myofibroblasts; neoangiogenesis sustained by vascular endothelial growth factor; and a maturation phase, regulated by matrix metalloproteinases and their tissue inhibitors, over which the scar becomes progressively more organized and stable [37,38]. The risk of developing it rises with the invasiveness of the index operation, fusion carrying a higher risk than microdiscectomy, with the number of previous operations, with advanced age and comorbidity such as diabetes or chronic inflammatory disease, and with inadequately controlled intraoperative bleeding.

The established scar produces four consequences within the epidural space: narrowing of the neural foramina, entrapment of the nerve roots, inflammation of the roots themselves, and venous engorgement from altered local blood flow [26,29]. Mechanical lysis is proposed to act on three of these. First, the scar tethers the nerve root to surrounding structures, abolishing the few millimeters of physiological excursion the root normally undergoes during flexion, extension, and straight-leg raising; a tethered root is repeatedly stretched rather than gliding, generating mechanical nociception with every movement. Advancing and rotating the catheter tip along the plane of least resistance is intended to separate these adhesions and restore that excursion. Second, the scar acts as a diffusion barrier: an injectate delivered from a distance simply flows around it, which is why an interlaminar or transforaminal steroid injection may fail in a patient whose epidural space has become compartmentalized [26,39]. By opening a channel to the target and injecting there, the catheter is intended to allow a drug that could not reach its site of action to do so; the epidurogram shows whether contrast reaches the target, the closest available surrogate for the drug. Third, fibrosis compresses the epidural venous plexus and impairs venous drainage, contributing to nerve root edema and to a locally ischemic environment; decompressing these compartments may allow venous outflow to resume. None of these three procedural effects—restored excursion, restored diffusion, restored venous outflow—has been measured directly in patients; they are inferred from the anatomy of the scar and from the change in contrast spread observed during the procedure.

The effect of mechanical lysis is therefore largely structural, and it should be greatest where adhesions are discrete and filmy. Where scar is dense, circumferential and mature—the typical picture in the previously operated spine—the catheter tip can create a channel but cannot dissolve the matrix around it. This is precisely the limitation that the enzymatic treatment component is intended to overcome.

#### 4.3.2. Hyaluronidase and Enzymatic Adhesiolysis

Hyaluronidase is an endoglycosidase that hydrolyzes the β-1,4 glycosidic bonds of hyaluronan, the glycosaminoglycan forming the hydrated ground substance in which the collagen fibers of scar tissue are embedded. Depolymerizing hyaluronan reduces the viscosity of this matrix and increases its permeability [17,30], with two consequences relevant to the epidural space. The first is chemical adhesiolysis in the strict sense: it is proposed that softening the ground substance loosens the collagen network, so that adhesions resisting the catheter tip become separable and lysis extends beyond the linear track the catheter can create mechanically. This proposed effect on established scar should not be assumed to be proven to the same extent as the spreading effect described next: direct evidence that epidural hyaluronidase meaningfully modifies mature fibrosis in patients is lacking. The second, and arguably the more important, is pharmacokinetic: by lowering the resistance of the interstitium, hyaluronidase markedly increases the volume of distribution of any agent co-administered with it, so that corticosteroid and local anesthetic spread more widely and reach nerve root surfaces that would otherwise remain shielded by scar [30,31]. This is the classical spreading-factor property for which the enzyme has been used in other fields of medicine for decades.

Because its action is enzymatic rather than mechanical, hyaluronidase is not limited by the geometry of the catheter track, which would be consistent with the observation in our clinical series (unpublished data, under review [13]) that the option including hyaluronidase was associated with substantial improvement in the most heavily scarred, previously operated patients—although that observation comes from a non-randomized series in which hyaluronidase was allocated precisely to those patients, and cannot test the hypothesis. The effect is transient, since hyaluronan is resynthesized; the enzyme is best understood as opening a temporary window of enhanced diffusion during which the co-administered drugs act, rather than as producing permanent structural change. A recombinant human preparation (Hylenex) was used in preference to animal-derived formulations, because it retains greater activity at physiological pH and avoids the immunogenicity associated with bovine or ovine enzyme [30,31]. It is generally well tolerated in the epidural space, the principal theoretical concerns being rare hypersensitivity reactions and the fact that the same increase in permeability that improves therapeutic spread would also accelerate the spread of an inadvertently misplaced injectate—one further reason why the anesthetic test dose precedes its administration.

#### 4.3.3. Pulsed Radiofrequency of the Dorsal Root Ganglion

**Figure 2:**
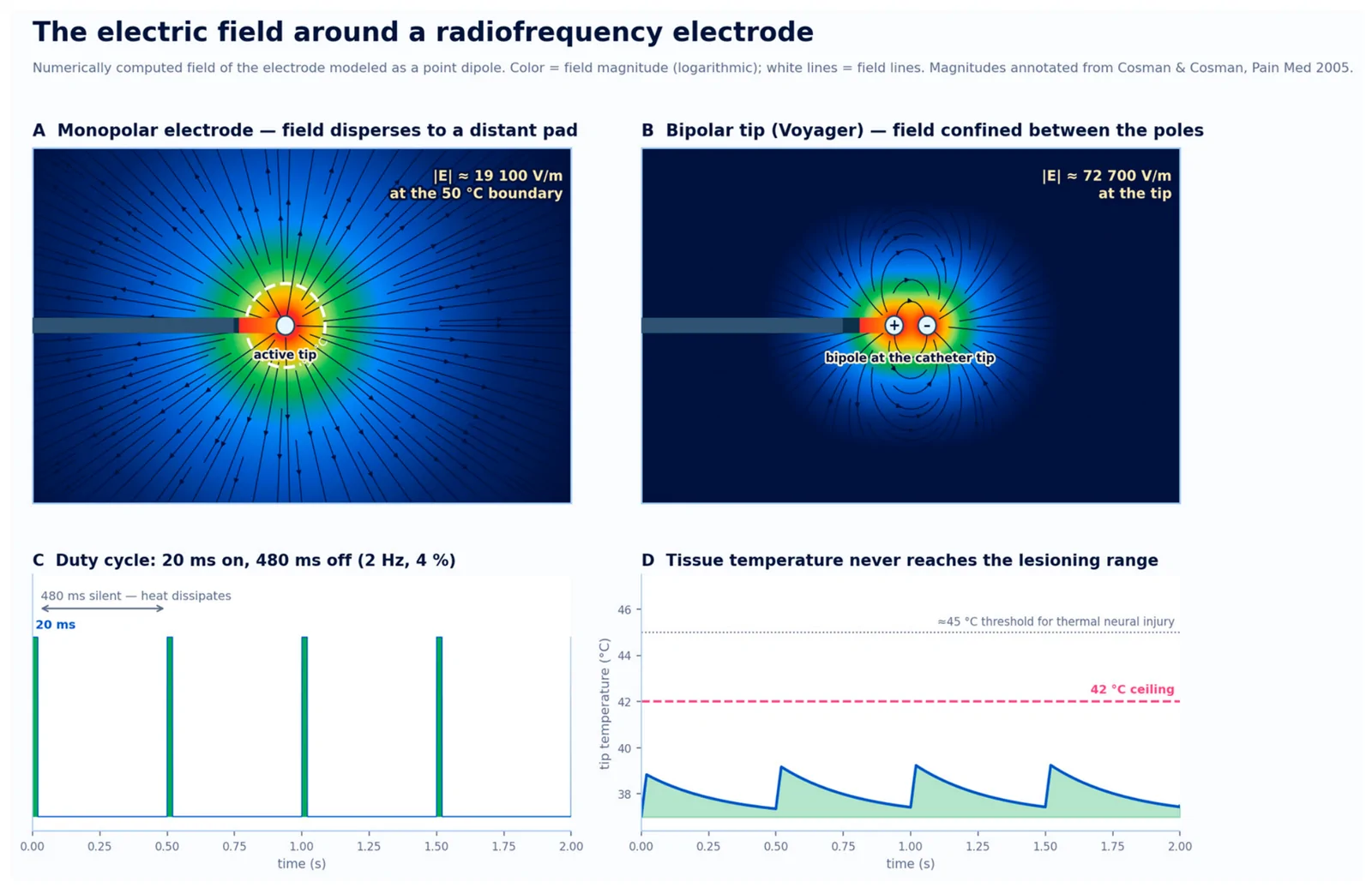
Continuous versus pulsed radiofrequency. Continuous radiofrequency produces a thermal lesion; pulsed radiofrequency delivers a peak electric field approximately four times higher at the electrode tip while the temperature ceiling of 42 °C prevents any lesion. Field magnitudes are the published model values of Cosman and Cosman [41]; the schematic is original. (**A**) Monopolar electrode: the field disperses toward a distant grounding pad; (**B**) bipolar tip: the field is confined between the two poles; (**C**) duty cycle of pulsed radiofrequency (20 ms on, 480 ms off); (**D**) tip temperature over successive pulses. Color scale: electric-field magnitude on a logarithmic scale, from blue (lowest) to red (highest); white lines with arrowheads: electric field lines; the white dashed circle in (**A**) marks the 50 °C boundary of a continuous-radiofrequency lesion. In (**D**), the red dashed line is the 42 °C ceiling of the generator and the dotted line the ≈45 °C threshold for thermal neural injury.

The dorsal root ganglion is an attractive target in neuropathic pain because it houses the cell bodies of primary sensory neurons, is the site at which ectopic discharge originates after nerve injury, and has a blood–nerve barrier that is relatively permeable. In continuous radiofrequency, current heats tissue above 60 °C and produces a coagulative lesion (Figure 2). When this is applied to a mixed sensory ganglion, there are risks of developing deafferentation pain and sensory loss. Pulsed radiofrequency was developed to separate the electrical effect from the thermal one. Current is delivered in short bursts—here 20 ms every 500 ms—so that the silent interval allows heat to dissipate and the tip temperature never exceeds 42 °C, well below the threshold for protein denaturation. What the tissue experiences is therefore not heat but a rapidly fluctuating electric field, most intense at the electrode tip.

**Figure 3:**
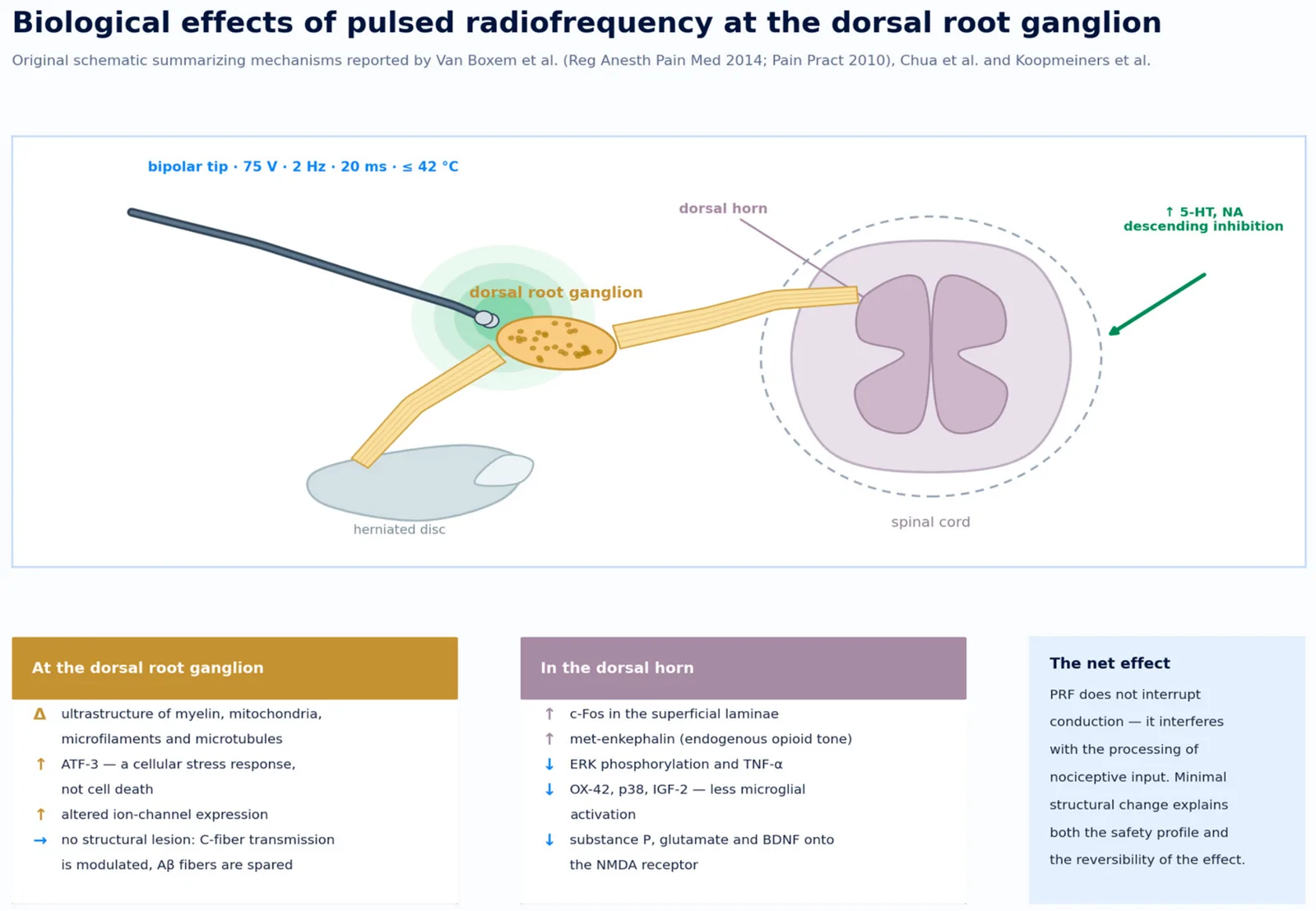
Biological effects of pulsed radiofrequency at the dorsal root ganglion and in the dorsal horn, summarizing the mechanisms reported by Van Boxem and colleagues and others [33,34,35,40]. The schematic is original. Symbols in the lower panels: Δ, structural change; ↑, increased; ↓, decreased; →, preserved (no structural lesion). The green halo marks the electric field around the bipolar tip; the green arrow indicates enhanced descending noradrenergic and serotonergic inhibition; the yellow structures are the dorsal root ganglion and its roots, the purple structure the spinal cord in cross-section.

Several mechanisms have been proposed for the resulting analgesia, and they probably act together (Figure 3). Exposure induces immediate early genes, notably c-Fos, in the superficial laminae of the dorsal horn [33,34,40], indicating that the intervention modifies activity in the second-order neurons with which the treated ganglion communicates. The field appears to depress excitatory synaptic transmission preferentially in small-diameter unmyelinated C-fibers, which carry nociceptive input, while sparing the larger myelinated fibers responsible for discriminative touch and proprioception—consistent with the clinical observation that pulsed radiofrequency reduces pain without producing numbness or motor deficit. Longer-term effects have been attributed to attenuation of central sensitization, to a shift in the local balance of pro- and anti-inflammatory cytokines, to epigenetic modulation of nociceptive signaling, and to enhanced activity in descending noradrenergic and serotonergic inhibitory pathways [5,14,35]. Ultrastructural changes in the mitochondria and microfilaments of C-fibers have also been described, suggesting a sublethal cellular stress response rather than the production of a lesion. These are preclinical observations: they establish that the field produces biological effects in neural tissue, but they do not by themselves demonstrate that any of them mediates the benefit observed in patients, and the relationship between the two remains an inference.

The spatial distribution of this field is decisive for the effect, and it has been characterized in detail by Cosman and Cosman [41], who modeled and measured the electric and thermal fields around radiofrequency electrodes. Two findings from that work apply directly to the technique used here. First, the electric field is far from uniform: it is greatest immediately at the electrode tip and falls very steeply with distance, so the biological effect of pulsed radiofrequency is concentrated within a small volume of tissue at the tip, and this effect depends critically on how close the electrode lies to the ganglion [41]. Second, whereas continuous radiofrequency produces a well-defined thermal lesion extending around the active tip, pulsed radiofrequency at a 42 °C ceiling produces only a minimal zone of tissue alteration at the very tip of the electrode—which is consistent with the clinical preservation of sensory and motor function. It follows that accurate positioning matters more in pulsed than in continuous radiofrequency, since a few millimeters of displacement move the target out of the region of high field intensity. This is one of the practical advantages of delivering the energy through a catheter that has already been steered to the target under fluoroscopic guidance.

Delivering the energy with a bipolar technique through the bipole at the catheter tip further defines and shapes the radiofrequency field. The current is confined between two poles a few millimeters apart rather than dispersing toward a distant grounding pad as in a monopolar arrangement, which produces a more predictable, better contained, and more intense field in the tissue between and immediately around the two poles. Because pulsed radiofrequency modifies neural signaling rather than removing a structural cause, its effect is expected to be reversible and time-limited—a prediction consistent with the progressive attenuation of benefit observed clinically after pulsed radiofrequency, whichever adjuncts are combined with it.

#### 4.3.4. Local Anesthetic and Corticosteroid: Rationale in Stenosis and Disc Herniation

The injection of a long-acting local anesthetic with a corticosteroid is not merely an adjunct: in the pathologies treated here, it addresses the inflammatory and neurochemical component of pain that mechanical and enzymatic lysis cannot reach. In lumbar disc herniation, the nucleus pulposus is an immunologically sequestered tissue [42]. When it contacts the epidural space, it provokes a chemical radiculitis mediated by phospholipase A2 (PLA2), TNF-α, IL-1β, and IL-6, which sensitizes the nerve root independent of any mechanical compression. This explains the familiar clinical paradox of severe radicular pain with only modest imaging findings, and it is the reason a substantial proportion of discogenic radicular pain responds to an anti-inflammatory intervention without any change in the size of the herniation.

Corticosteroids act at several points of this cascade. They inhibit PLA2 and thereby the generation of arachidonic-acid metabolites, suppress the transcription of pro-inflammatory cytokines, reduce capillary permeability and root and perineural edema, and stabilize neuronal membranes with a direct suppressive effect on ectopic discharge from injured C-fibers. In the stenotic and post-surgical spine, the rationale is complementary rather than identical [32]: reducing inflammatory edema within a canal or foramen whose bony dimensions are fixed produces a disproportionate gain in effective cross-sectional area, since in a non-compliant compartment even a small reduction in soft-tissue volume translates into a marked decrease in pressure on the root.

The local anesthetic contributes in three ways that go beyond immediate comfort. It provides diagnostic confirmation, since abolition of the patient’s typical pain distribution after injection at the target verifies that the correct root has been treated. It interrupts nociceptive input at the moment of maximal mechanical stimulation, and this transient blockade of afferent barrage during and immediately after lysis may reduce the wind-up of dorsal horn neurons that would otherwise follow the procedure. Finally, sodium-channel blockade suppresses the ectopic discharge that characterizes injured and sensitized fibers, an effect that in neuropathic pain frequently outlasts the pharmacological duration of the drug. Levobupivacaine was selected for its long duration of action and its more favorable cardiac and central nervous system safety profile compared with racemic bupivacaine, an advantage that matters in an elderly population and when a substantial epidural volume is injected.

The combination is therefore rational in mechanistic terms, and its components are complementary: the catheter restores access, hyaluronidase widens the distribution of what is injected, corticosteroid and anesthetic act on inflammation and neuronal excitability once they reach the root, and pulsed radiofrequency modulates the ganglion in which the sensitized primary sensory neuron cell bodies reside.

### 4.4. Available Techniques and Description of the Combined Procedure

#### 4.4.1. From the Original Racz Description to the Multifunctional Catheter

Percutaneous epidural adhesiolysis was introduced by Gabor Racz in the 1980s [43,44], following the observation that patients with persistent radicular pain after spine surgery frequently showed, on epidurography, filling defects corresponding to the symptomatic root, and that conventional epidural injections failed to reach that root. The original technique used a spring-wound, radiopaque catheter introduced through the sacral hiatus and advanced to the affected level, where mechanical lysis was combined with the injection of hypertonic saline, hyaluronidase, and corticosteroid, classically over a protocol spread across three consecutive days. The conceptual innovation was not the drug but the target: the procedure treated the scar that prevented drug delivery, rather than the inflammation alone.

The classical technique as currently described—caudal entry with a Coudé needle, epidurography, navigation of a spring-wound catheter to the filling defect, hyaluronidase, local anesthetic with corticosteroid after a test dose and an observation interval for subdural spread, and finally hypertonic saline, repeated over one to three days—is set out step by step in Section 4.4.2.

The procedure described here stands in direct continuity with that technique while differing from it in four respects: a two-day rather than a three-day protocol; the omission of hypertonic saline; a lower hyaluronidase dose, 900 units combined with betamethasone rather than 1500 units with methylprednisolone acetate; and the integration of pulsed radiofrequency delivered through the same multifunctional catheter, which the classical technique does not utilize. The retention of an indwelling catheter for a single night, allowing a second pharmacological administration without a further puncture, is inherited directly from the original description.

#### 4.4.2. The Classical Racz Technique, Balloon-Inflatable Catheters and Epiduroscopy

Percutaneous epidural adhesiolysis is not a single procedure but a family of techniques (Table 2) that share an objective—restoring access to a nerve root shielded by scar—while differing in how the scar is engaged, in what is instilled, in whether the epidural space is visualized directly, and in how long the catheter remains in place. The differences matter clinically because they map onto different indications, and in particular onto the distinction between persistent spinal pain syndrome type 1 and type 2.

The original description remains the reference against which the others are judged. A 15- or 16-gauge radiopaque Coudé needle is introduced through the sacral hiatus at about 45 degrees to the sacrococcygeal membrane and then reduced to roughly 30 degrees; an epidurogram with approximately 10 mL of non-ionic water-soluble contrast establishes the normal branching pattern and identifies filling defects; a spring-wound catheter, its tip bent about 30 degrees some 2.5 cm from the end, is navigated to the affected root; 1500 units of hyaluronidase in 10 mL of preservative-free saline are injected, followed by a 3 mL test dose of local anesthetic with 80 mg methylprednisolone acetate and, after five minutes, the remainder; a further twenty-minute interval is intended to reveal subdural spread, which characteristically declares itself only after 16 to 18 min; and 10 mL of 10% hypertonic saline is then infused over 30 min. The sequence is repeated over one, two, or three days according to the protocol chosen, and antibiotic prophylaxis is continued while the catheter remains in situ [26,43,44].

Two elements of the classical technique have become contentious. Hypertonic saline, introduced for its osmotic effect on the edematous root, is omitted in many contemporary practices because of the consequences should it reach the intrathecal space, and its incremental benefit over the mechanical and enzymatic components has never been isolated. Multi-day protocols with an indwelling catheter, likewise, are increasingly compressed into one or two days to limit the duration of indwelling access. The technique reported here retains the caudal approach, the epidurographic endpoint, the enzymatic component, and a single overnight catheter, but omits hypertonic saline and adds neuromodulation.

A different mechanical principle is offered by the balloon-inflatable catheter, of which the ZiNeu device (KLN, Hong Kong, China) is the best described. Rather than separating adhesions by advancing and rotating a tip, the catheter carries a balloon that is inflated within the ventral epidural space or at the foraminal entrance, so that the dilating force is radial rather than linear. Choi et al. [23] described its use for percutaneous adhesiolysis and decompression and reported that it could be advanced to target levels that a conventional catheter had failed to reach, with decompression achieved in patients with foraminal and central stenosis.

The rationale differs from that of the steerable catheter in an instructive way. A steerable tip creates a channel and a balloon widens a compartment. Where the dominant problem is a filmy adhesion tethering the root, the first is sufficient. Where the foramen is narrowed, or a compartment will not accept volume, radial dilation may achieve what linear advancement cannot. The two are therefore complementary rather than competing, and the choice follows the epidurogram: a defect that resolves with catheter advancement calls for the former, one that persists despite the tip having reached the level may call for the latter.

Epiduroscopy adds direct vision. A flexible fiber-optic endoscope introduced through the sacral hiatus allows the epidural space to be inspected, adhesions to be identified visually and lysed under direct control, and the appearance of the root—inflamed, tethered, encased in scar—to be documented rather than inferred from a contrast pattern. Its advantages are diagnostic as much as therapeutic, as it distinguishes fibrosis from other causes of a filling defect and confirms that lysis has been achieved at the intended site.

Its costs are equally clear. The equipment and the learning curve are substantially greater; the procedure is longer; the volumes of irrigation required carry a risk of raised intraspinal and intraocular pressure; and access is limited in a severely stenotic canal. In the algorithm of Van Boxem and colleagues, epiduroscopy and adhesiolysis appear together at the end of the pathway and with the caveat that they be performed in specialized centers [32]. Epiduroscopy is therefore best regarded as the option for the diagnostically uncertain or repeatedly failed case rather than as a first-line alternative to catheter-based lysis, a position consistent with the recent Italian Delphi consensus on endoscopic epidurolysis [45].

#### 4.4.3. The Catheter and the Three Treatment Options

All patients were treated through the same multifunctional steerable epidurolysis catheter (AlfaMed—Voyager), introduced via the sacral hiatus. The device integrates in a single instrument the three functions that would otherwise require separate devices: mechanical lysis of adhesions by controlled advancement and steering of the tip; injection of contrast medium and therapeutic agents through its lumen; and delivery of radiofrequency energy. The catheter carries a bipole at its tip, which allows bipolar pulsed radiofrequency of the dorsal root ganglion to be performed through the catheter itself, with the current confined between the two closely spaced poles rather than dispersed toward a remote grounding pad. Because the catheter is navigated to the target foramen under fluoroscopic and contrast control, the electrode is positioned exactly where the obstruction was demonstrated, and neuromodulation can be delivered without exchanging devices or performing a second puncture.

**Figure 4:**
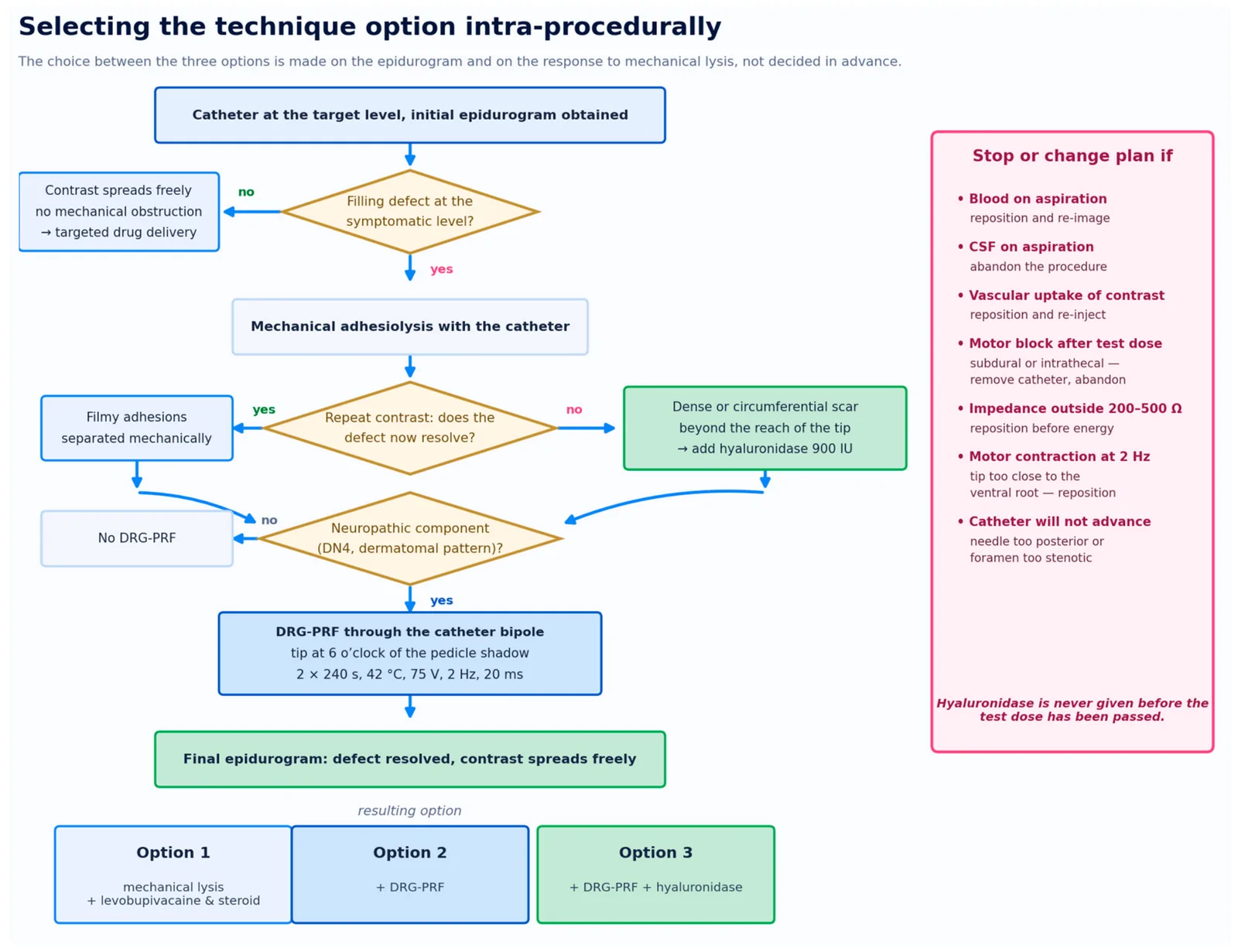
Selecting the technique option intra-procedurally. The choice between the three options follows the epidurogram and the response to mechanical lysis rather than being decided in advance; the panel on the right lists the findings that require the plan to be changed or the procedure abandoned. The algorithm reflects the practice of the authors’ center and has not been validated in comparative studies. Arrows indicate the sequence of steps; diamonds are decision points, with the answer (yes/no) written beside the corresponding arrow; the three boxes at the bottom are the resulting treatment options; the panel on the right is read independently of the flow.

According to the underlying pathology and the intra-procedural findings, the operator selects one of three options; the decision follows the epidurogram and the response to mechanical lysis rather than being fixed in advance (Figure 4). This is an institutional procedural algorithm derived from the operator’s practice and from the anatomical reasoning set out in Section 4.3; it has not been validated in comparative studies, and the three options have never been compared with each other under randomized allocation:

- Option 1—mechanical adhesiolysis with pharmacological injection. Mechanical lysis of epidural adhesions with the catheter alone, followed by injection of long-acting levobupivacaine combined with methylprednisolone acetate 40 mg.
- Option 2—mechanical adhesiolysis, pharmacological injection and DRG-PRF. Epidurolysis and injection of the same drug combination, with the addition of pulsed radiofrequency of the dorsal root ganglion.
- Option 3—mechanical adhesiolysis, DRG-PRF and enzymatic adhesiolysis. Epidurolysis, DRG-PRF, and injection of recombinant human hyaluronidase 900 IU with betamethasone 8 mg in a total volume of 5 mL, for enzymatic lysis of established scar.

#### 4.4.4. Pulsed Radiofrequency Parameters

Where pulsed radiofrequency was applied (Options 2 and 3), it was delivered through the bipole at the catheter tip positioned at the dorsal root ganglion, using the following parameters:

- Mode: bipolar pulsed radiofrequency, delivered between the two poles at the catheter tip.
- Pulse frequency: 2 Hz.
- Pulse width: 20 ms—a 4% duty cycle, leaving a 480 ms silent phase for heat dissipation.
- Maximum tip temperature: 42 °C, continuously monitored and automatically limited by the generator, keeping tissue below the ≈45 °C threshold for thermal neural injury so that the effect is neuromodulatory rather than ablative.
- Output voltage: 75 V, automatically titrated downwards by the generator whenever the 42 °C ceiling is approached before the end of the cycle. At this output, the temperature limit, rather than the voltage setting, is the effective constraint on energy delivery.
- Duration: two consecutive cycles of 240 s (480 s in total) at each treated ganglion.
- Impedance: measured before each cycle and required to fall within 200–500 Ω. Values below this range suggest a short circuit or intravascular position, and values above it suggest poor tissue contact or an insulating layer of scar; in either case, the catheter was repositioned before energy was delivered.
- Stimulation testing: sensory stimulation at 50 Hz reproducing the patient’s usual paresthesia in the target dermatome at ≤0.5 V confirmed proximity to the ganglion; motor stimulation at 2 Hz verified the absence of segmental muscle contraction at twice the sensory threshold before energy delivery.

The choice between the three options follows the dominant pathology and the intra-procedural appearance of the epidural space rather than a randomization schedule.

#### 4.4.5. Interventional Technique, Step by Step

**Figure 5:**
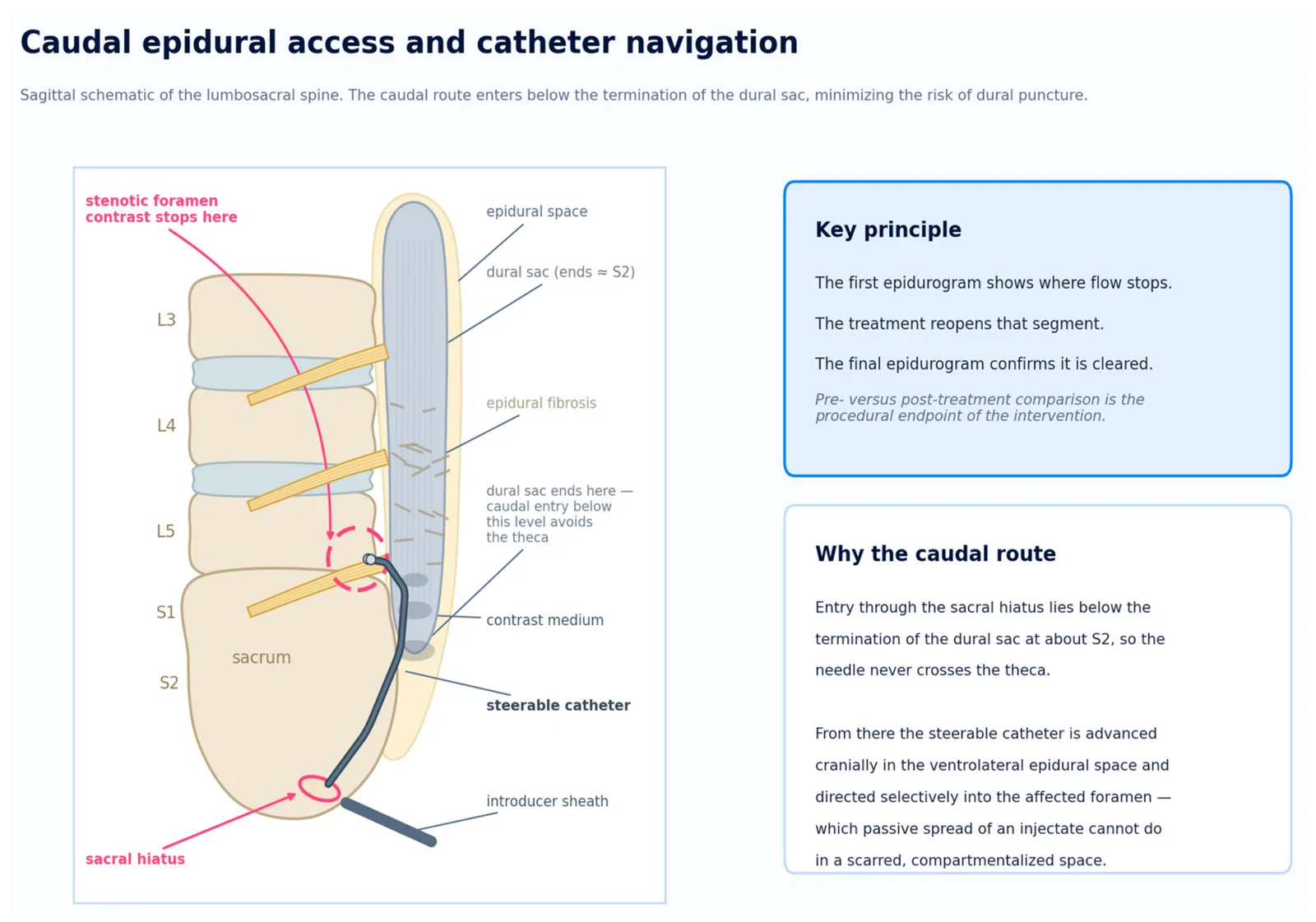
Caudal epidural access and catheter navigation. The caudal route enters the epidural space below the termination of the dural sac, minimizing the risk of dural puncture. The catheter is advanced cranially in the ventrolateral epidural space to the stenotic foramen at which contrast flow stops.

The technique used in this series is built around a single steerable epidural catheter introduced through the sacral hiatus, which serves simultaneously as a mechanical instrument, a diagnostic tool, and a drug-delivery device. The caudal route was chosen deliberately (Figure 5): it enters the epidural space below the termination of the dural sac, which minimizes the risk of inadvertent dural puncture, and it allows the catheter to be advanced cranially along the ventrolateral epidural space toward the affected root under continuous fluoroscopic control. Because the catheter is steerable, its tip can be directed selectively into the target foramen rather than relying on the passive spread of an injectate, which is precisely the limitation of conventional interlaminar or transforaminal injections in a scarred epidural space.

The logic of the sequence is diagnostic before therapeutic. The initial epidurogram is not a formality but the pivotal step of the procedure. Magnetic resonance imaging and computed tomography have a reported sensitivity and specificity of only 50% to 70% for epidural fibrosis [39], and neither identifies scar tissue with complete reliability, whereas epidurography demonstrates filling defects directly [29,39] and shows in real time where injected fluid stops. It therefore converts an anatomical hypothesis derived from imaging into a functional demonstration of impaired spread. A filling defect is not, however, specific for symptomatic epidural fibrosis or for the pain-generating level: it may reflect asymptomatic scar, a technical cause, or simply the anatomy of a stenotic foramen. It is interpreted together with the patient’s clinical presentation and the pre-procedural imaging, and it is a defect that is concordant with the dermatomal distribution of the pain and with the imaging findings that defines the segment to be treated—the segment in which any previously injected drug would, on the same reasoning, have failed to reach its target. The lidocaine test dose that follows serves an entirely different purpose—it is a safety checkpoint. By confirming the expected pattern of sensory change in the lower limbs and the absence of rapid-onset dense motor block, it helps to identify intrathecal placement, while the absence of systemic symptoms argues against intravascular positioning; neither check is an absolute guarantee, and both reduce rather than abolish the risk. No therapeutic agent, and in particular no hyaluronidase or corticosteroid, is administered before this checkpoint has been passed.

**Figure 6:**
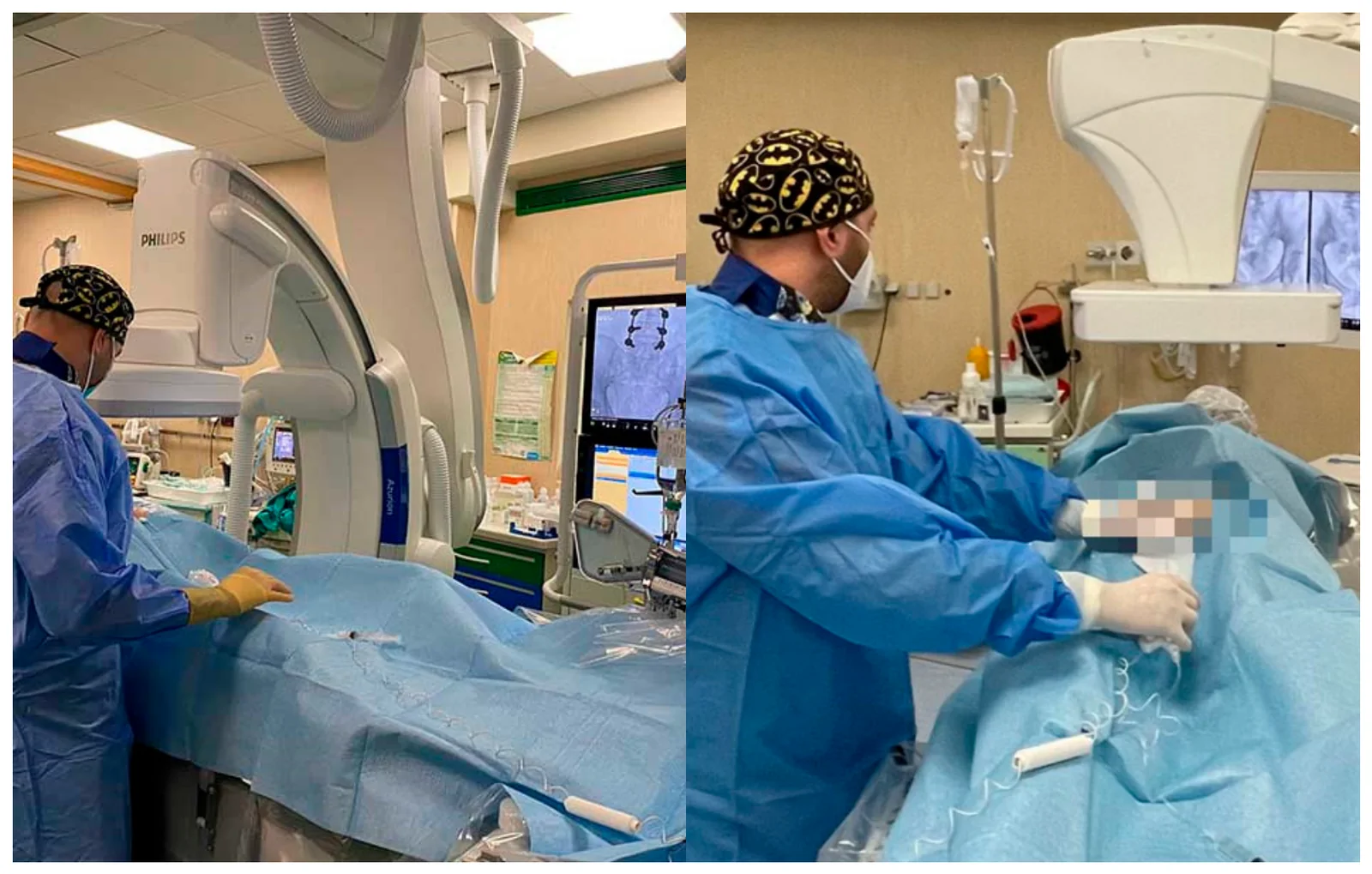
Mechanical adhesiolysis in progress. The operator advances and rotates the steerable catheter under intermittent fluoroscopic control while observing the live image; catheter position is adjusted until the tip reaches the target foramen. Patient-identifying detail has been obscured.

Adhesiolysis itself is then performed in a graded fashion (Figure 6). Mechanical lysis relies on the controlled advancement and rotation of the catheter tip to separate adhesions along the plane of least resistance; this is effective for filmy, discrete bands but is limited where scar is dense and circumferential, as is typically the case in previously operated patients. Hyaluronidase is intended to extend the reach of the procedure by hydrolyzing the hyaluronan-rich ground substance of epidural fibrosis, softening scar that the catheter tip cannot mechanically disrupt and thereby improving the diffusion of the co-administered agents. Where pulsed radiofrequency is added, the electrode is positioned at the dorsal root ganglion, and two cycles of 240 s are delivered at a controlled tip temperature of 42 °C, remaining below the threshold for thermal neural injury so that the effect is neuromodulatory rather than lesional. The final contrast injection closes the diagnostic loop opened by the first: comparison of the pre- and post-treatment epidurograms provides immediate, objective confirmation that the previously obstructed segment has been reopened and constitutes the procedural endpoint of the intervention.

Throughout, the operator maintains a direct line of sight to the fluoroscopic monitor, and radiation exposure is limited by the use of intermittent pulsed screening rather than continuous fluoroscopy, with the C-arm draped sterilely and positioned to obtain antero-posterior and oblique projections without breaking the sterile field.

**Figure 7:**
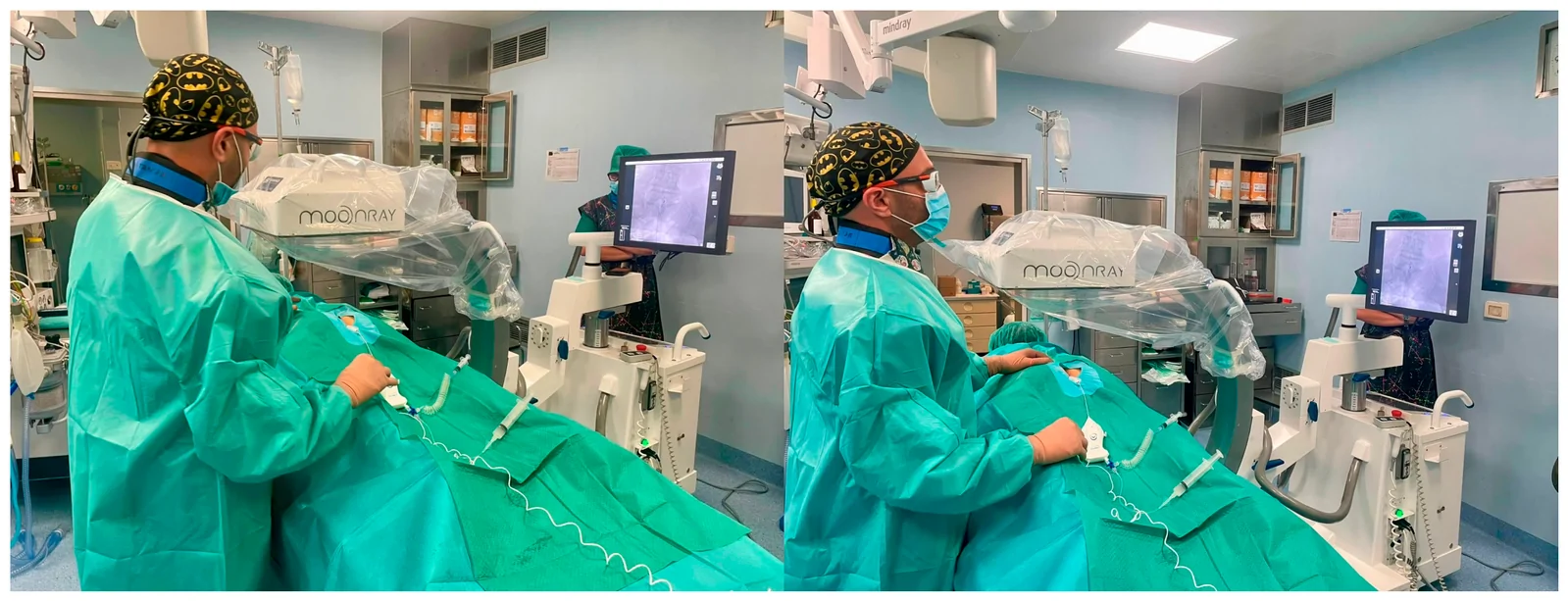
Procedural set-up in the operating room. The patient is positioned prone and fully draped; the mobile C-arm (Moonray) is covered with a sterile drape and positioned over the lumbosacral region, with the live fluoroscopic image displayed on the monitor at the operator’s eye level. The operator works from the patient’s left, maintaining a direct line of sight to the screen throughout catheter navigation.

**Figure 8:**
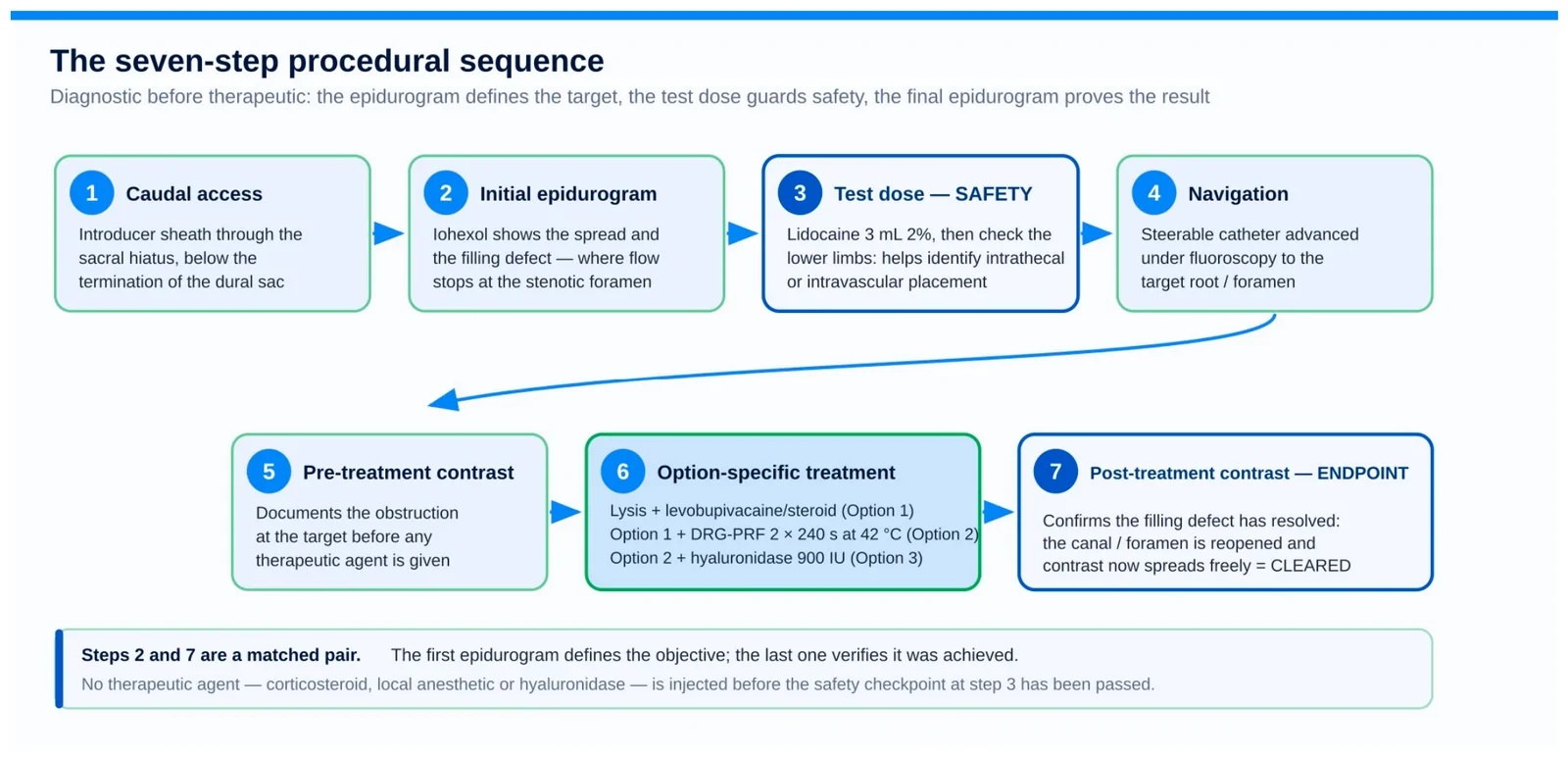
The seven-step procedural sequence.

All procedures were performed under fluoroscopic guidance with the patient prone, under aseptic conditions and with continuous hemodynamic monitoring (Figure 7). The sequence is summarized in Figure 8. Antibiotic prophylaxis with cefazolin 2 g intravenously was administered 30 min before skin puncture, unless a beta-lactam allergy was documented, in which case clarithromycin was substituted. Prophylaxis is not universally applied to single-shot epidural injections, but it is justified here because the catheter is left in situ for a second administration on the following day: the determinant of infective risk in this protocol is the duration of indwelling access rather than the puncture itself. The procedure then followed the standardized sequence set out below.

**Figure 9:**
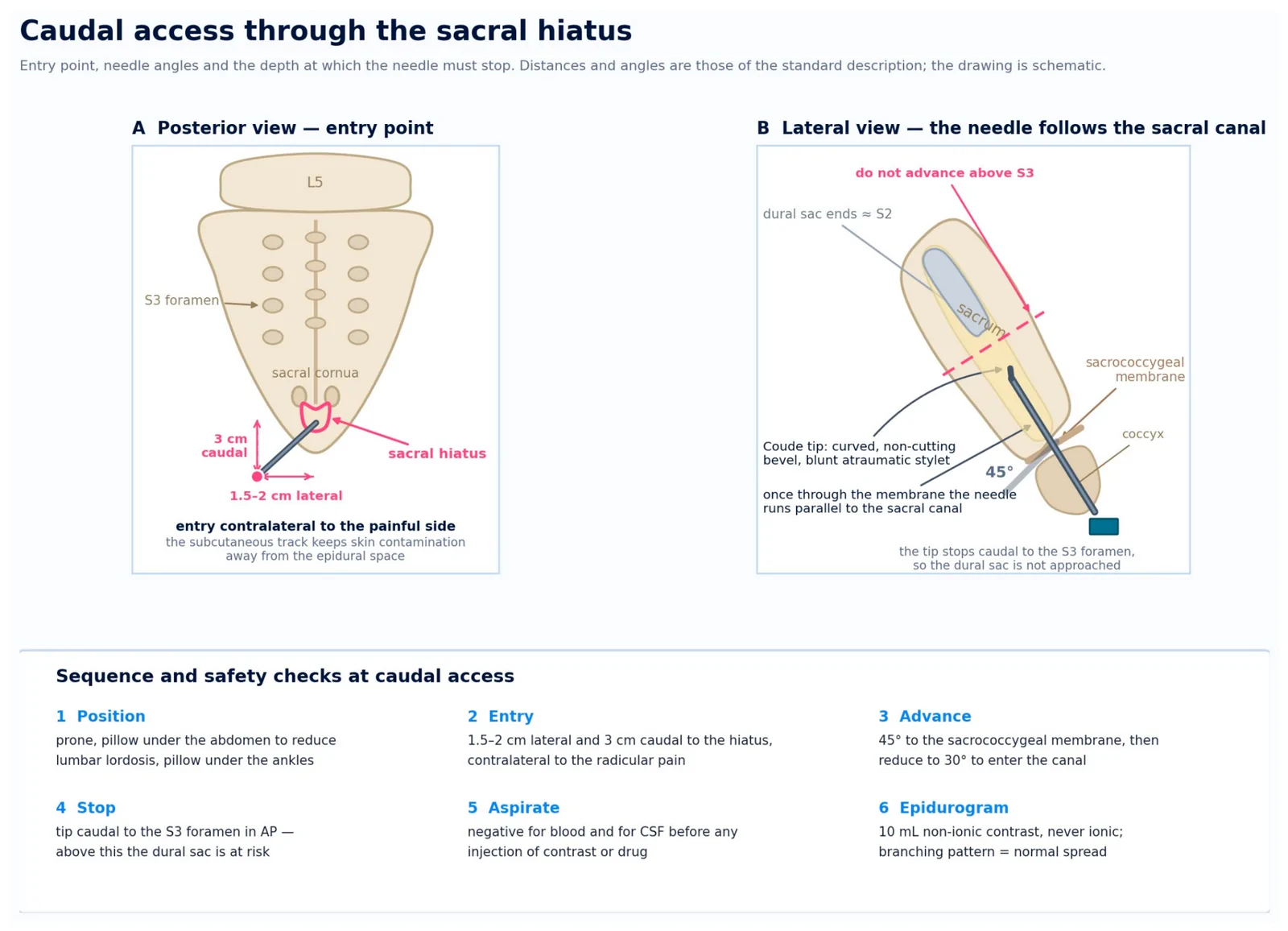
Caudal access through the sacral hiatus. (**A**) Posterior view showing the entry point 1.5–2 cm lateral and 3 cm caudal to the hiatus, contralateral to the painful side. (**B**) Lateral view showing the two needle angles and the level at which advancement must stop. The lower panel summarizes the sequence and the safety checks.

**Figure 10:**
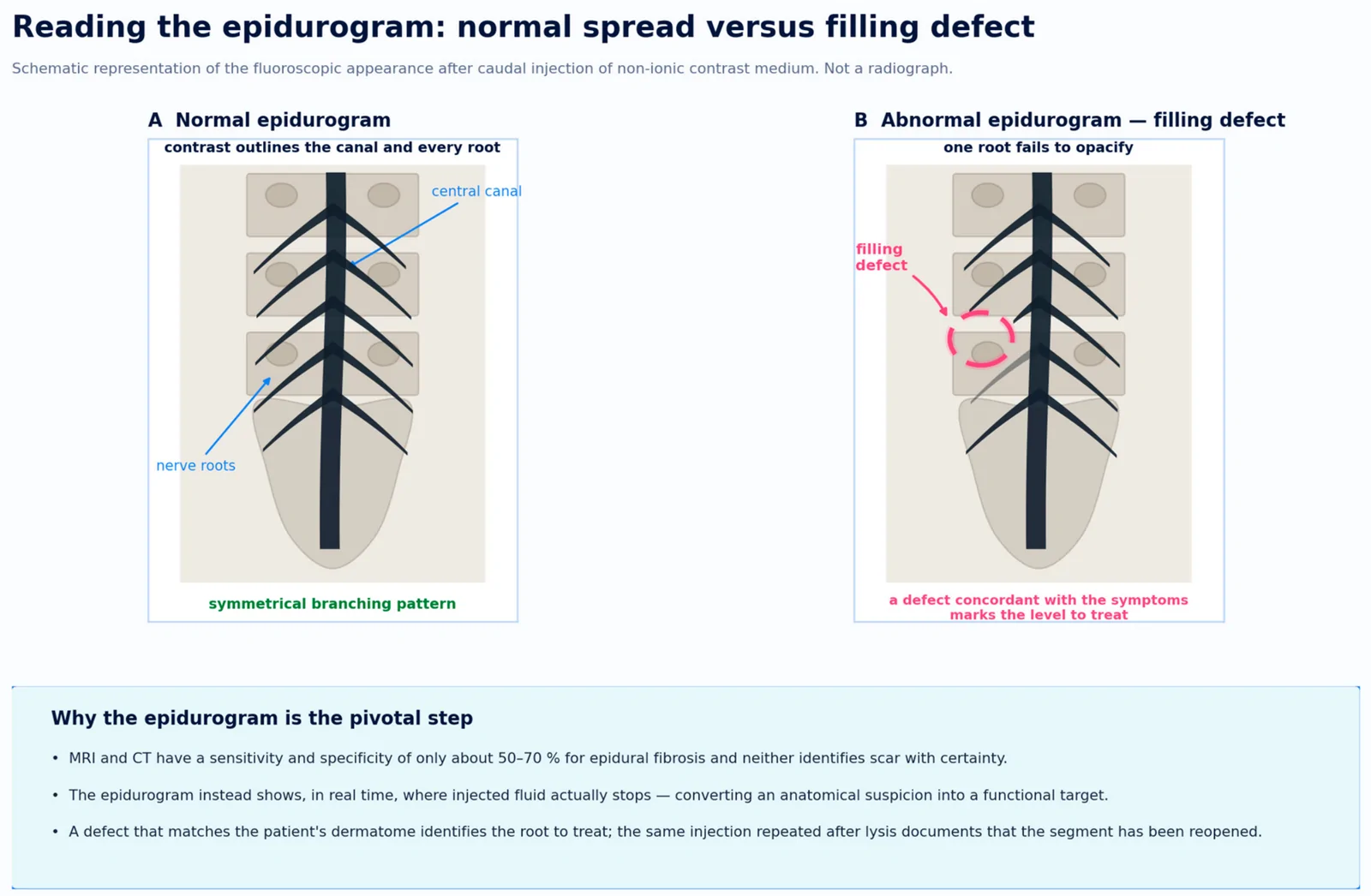
Reading the epidurogram. (**A**) Normal spread: contrast fills the canal and outlines every root, producing the symmetrical branching pattern. (**B**) Abnormal study: one root fails to opacify, and the filling defect marks the level at which flow is obstructed. Comparison of this image with the one obtained after treatment constitutes the procedural endpoint.

**Figure 11:**
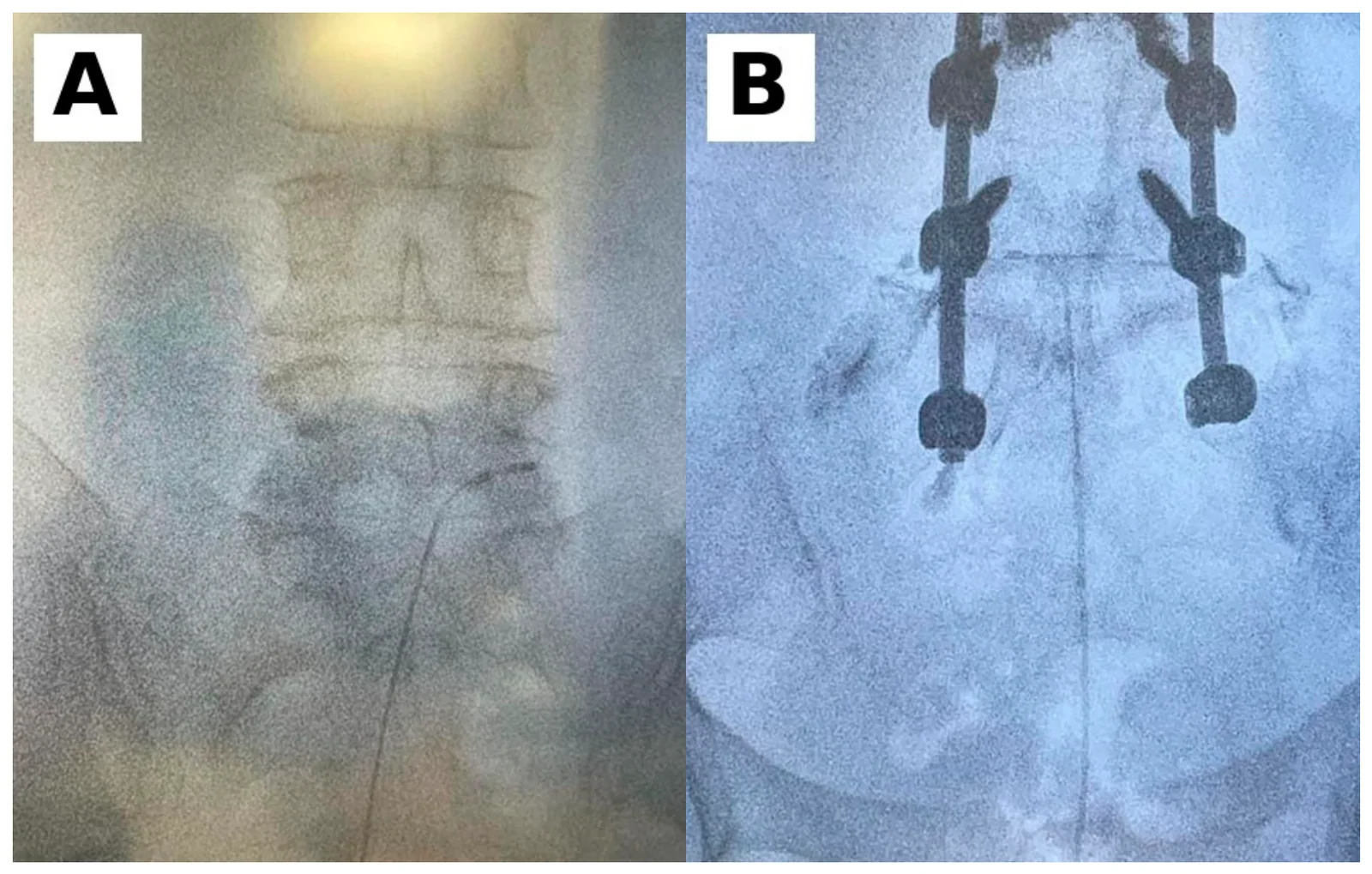
Epidurograms obtained during the procedure (antero-posterior fluoroscopy). (**A**) Epidurolysis with dorsal root ganglion pulsed radiofrequency: the radiopaque catheter has been advanced to the target level, and the contrast outlines the treated segment, with a symmetrical branching pattern once the root has been reached. (**B**) The same procedure with the addition of the enzymatic component in a previously operated spine with posterior instrumentation: hyaluronidase has been injected through the catheter at the target after mechanical lysis, and the contrast distribution after treatment documents that the previously obstructed segment has been reopened. These are the real-image counterparts of the schematic patterns in Figure 10.

**Figure 12:**
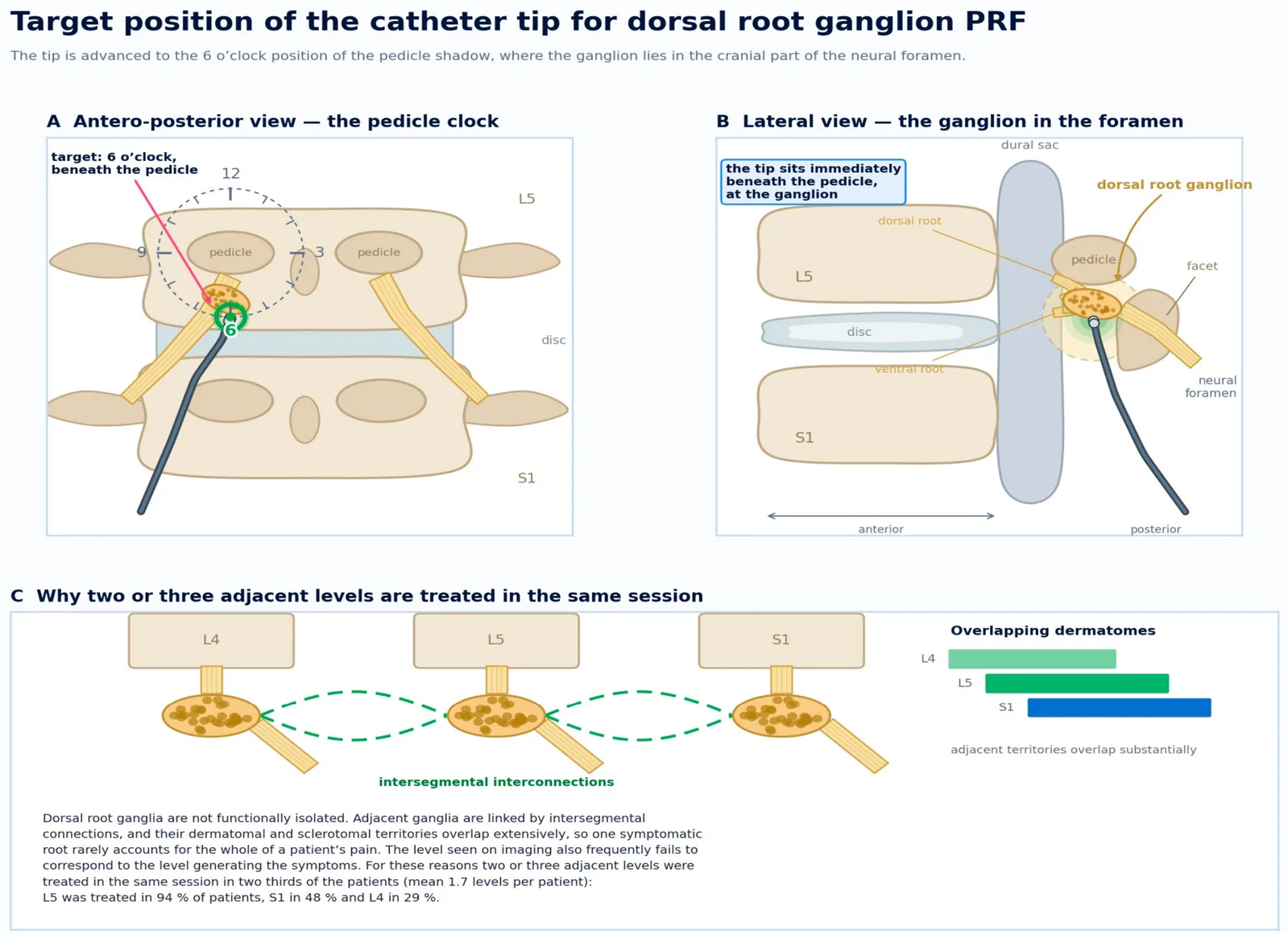
Target position of the catheter tip for dorsal root ganglion pulsed radiofrequency. (**A**) Antero-posterior view: the tip is placed at the 6 o’clock position of the pedicle shadow. (**B**) Lateral view, anterior to the left: the dural sac lies posterior to the vertebral bodies within the spinal canal, the dorsal and ventral roots leave it to enter the neural foramen, and the ganglion lies in the cranial part of the foramen, immediately beneath the pedicle. (**C**) Intersegmental interconnections and dermatomal overlap explain why two or three adjacent levels are usually treated. Colors: bone, beige; intervertebral disc, blue-grey; dural sac, grey; nerve roots and ganglia, yellow; catheter, dark grey with the bipolar tip in green. In (**A**), the dashed circle is the pedicle “clock” and the green marker the 6 o’clock target; in (**B**), the double-headed arrow gives the anterior–posterior orientation; in (**C**), the dashed green lines indicate intersegmental connections and the horizontal bars the overlapping dermatomal territories of L4, L5 and S1.

1. Caudal access. The caudal epidural space was entered through the sacral hiatus using a dedicated introducer sheath. The skin was punctured approximately 1.5 to 2 cm lateral and 3 cm caudal to the hiatus, contralateral to the side of the radicular pain, so that the subcutaneous track keeps any skin contamination away from the epidural space. The needle was advanced at about 45 degrees until contact with the sacrococcygeal membrane (Figure 9), and the angle was then reduced to about 30 degrees to enter the canal, the tip being kept caudal to the S3 foramen on the antero-posterior projection so that the dural sac, which terminates at approximately S2, is not approached. Negative aspiration for blood and for cerebrospinal fluid was confirmed before any injection; aspiration reduces, but does not eliminate, the risk of an unrecognized intravascular or intrathecal position.
2. Initial epidurogram. Non-ionic iodinated contrast medium (iohexol) was injected slowly under continuous fluoroscopy to visualize epidural spread and to identify filling defects or obstruction at the target level. Ionic contrast media are not used because inadvertent subarachnoid injection of an ionic agent can produce severe neurological complications. The interpretation of contrast spread patterns, and the distinction between accurate epidural placement and the appearances that signal misplacement, has been reviewed in detail elsewhere. A normal epidurogram shows a symmetrical branching pattern (Figure 10; for the corresponding fluoroscopic appearance before and after treatment see Figure 11) [39], the central canal forming the trunk and the outlined nerve roots the branches; an abnormal study shows areas into which contrast does not diffuse. These filling defects indicate the presumed location of scar tissue; they are treated as the target only when they are concordant with the patient’s radicular distribution and with the pre-procedural imaging, since a filling defect on its own is not specific for symptomatic fibrosis.

3. Anesthetic test dose and neurological check. A test dose of 3 mL of lidocaine 2% (60 mg) was administered through the catheter, and the lower limbs were checked over the following minutes for the onset of numbness or motor block. The expected pattern—segmental sensory change without rapid-onset dense motor block—supports an epidural position; a dense or rapidly ascending block, or systemic symptoms, indicates intrathecal, subdural, or intravascular spread. The test dose helps to identify misplacement and reduces the risk of injecting a therapeutic agent into the wrong compartment, but it does not exclude it with certainty, which is why the observation interval and the subsequent contrast controls are retained.
4. Catheter navigation to the target. The steerable catheter was advanced under fluoroscopic control to the target nerve root or foramen. For radiofrequency treatment, the tip was positioned beneath the pedicle, at the 6 o’clock position of the pedicle shadow on the antero-posterior projection, which corresponds to the cranial part of the neural foramen where the dorsal root ganglion lies (Figure 12). This position places the bipole immediately adjacent to the ganglion—where the electric field is most intense—while keeping it away from the disc anteriorly and caudally. Correct placement was confirmed by sensory stimulation at 50 Hz reproducing the patient’s usual paresthesia at ≤0.5 V and by the absence of segmental motor contraction on stimulation at 2 Hz.

Because dorsal root ganglia are not functionally isolated—adjacent ganglia are linked by intersegmental connections and their dermatomal and sclerotomal territories overlap extensively—a single symptomatic root rarely accounts for the whole of a patient’s pain, and the level identified on imaging frequently does not correspond exactly to the level generating the symptoms. For these reasons, two or three adjacent levels are commonly treated in the same session at our center: in our clinical series (unpublished data, under review [13]), two-thirds of the patients received treatment at more than one level, with a mean of 1.7 levels per patient. This is reflected in the anatomical distribution of that series, in which L5 was treated in 94% of patients, S1 in 48% and L4 in 29%, so that most patients received treatment at two or more contiguous levels. This multilevel treatment is current practice rather than an evidence-based requirement: the frequency with which it is performed does not establish that it is superior to single-level treatment, and no study has compared the two.

5. Pre-treatment contrast control. Contrast injected at the target documented the pre-treatment obstruction—the level at which flow was blocked by the stenotic canal or foramen.
6. Treatment. The option-specific treatment was then delivered: mechanical adhesiolysis with the catheter, with or without DRG-PRF, together with the option-specific pharmacological or enzymatic injection.
7. Post-treatment contrast confirmation. A final contrast injection confirmed resolution of the filling defect (Figure 11)—that the previously obstructed canal or foramen had been reopened and that contrast now spread freely across the treated segment. This radiological confirmation of a “cleared” epidural space is the procedural endpoint of the intervention.

#### 4.4.6. Preparation and Delivery of the Injectate

**Figure 13:**
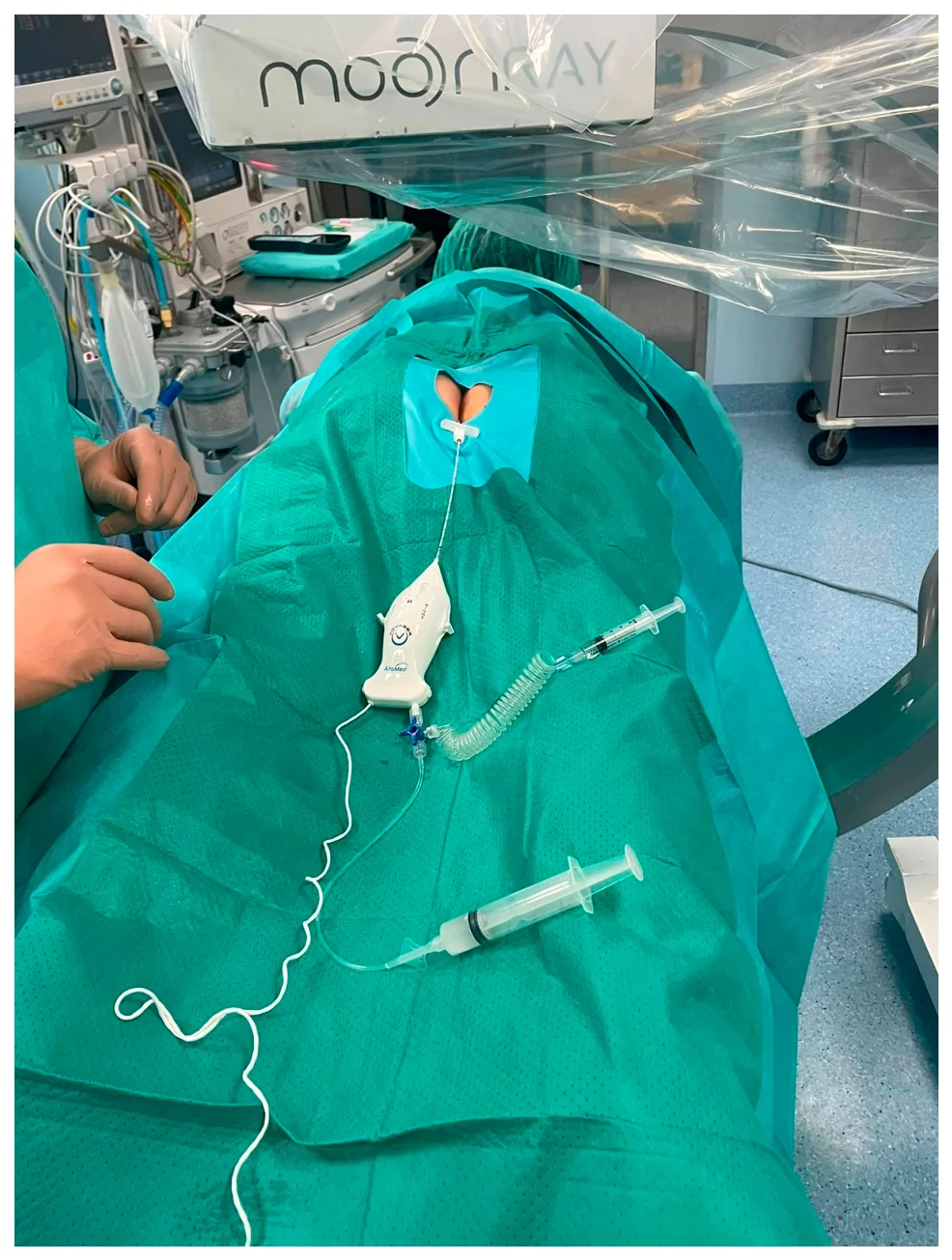
Close-up of the caudal access. The introducer sheath is secured at the sacral hiatus, and the steerable multifunctional catheter emerges through its hemostatic valve. The small syringe contains the non-ionic contrast medium and is connected by a coiled extension line to a three-way stopcock on the catheter, so that contrast can be injected at any point of the procedure without disconnecting the system. The 20 mL syringe contains the therapeutic mixture for the whole session: long-acting local anesthetic (levobupivacaine 2.5 mg/mL, 10 mL), methylprednisolone acetate 40 mg (1 mL), and normal saline 9 mL, for a total volume of 20 mL that is divided among the levels treated during the session, with a fractionated injection at each target (Section 4.4.6). The stopcock allows the operator to alternate between contrast and therapeutic injection and to steer and advance the catheter, without repositioning the sheath.

The injection system was assembled before the procedure so that diagnostic and therapeutic injections could be alternated without ever disconnecting it. A small syringe containing the non-ionic contrast medium was connected through a coiled extension line to a three-way stopcock mounted on the hub of the multifunctional catheter (Figure 13). This arrangement allowed contrast to be injected at any point—for the initial epidurogram, for the pre-treatment control at the target, and for the final confirmation—while the operator continued to steer and advance the catheter, and it avoided the repeated disconnections that would otherwise risk contaminating the system or displacing the tip.

Where hyaluronidase is not used (Options 1 and 2), the therapeutic mixture was prepared in a 20 mL syringe connected to the second port of the same stopcock and consisted of levobupivacaine 2.5 mg/mL, 10 mL; methylprednisolone acetate 40 mg (1 mL); and normal saline, 9 mL, for a final volume of 20 mL (levobupivacaine 1.25 mg/mL). The whole 20 mL was administered, but not at a single point: it was divided between the levels treated in that session, with fractionated injection at each target after the catheter had been repositioned and the new position confirmed with contrast. The dilution serves two purposes: it provides a volume sufficient to reach several adjacent foramina—necessary because two or three levels are routinely treated—while keeping the anesthetic concentration low enough to produce sensory rather than dense motor blockade, so that the neurological check performed after the test dose remains interpretable throughout the procedure.

In Option 3, the injectate differed because the objective was enzymatic lysis of established epidural scar rather than periradicular anti-inflammatory delivery. These patients received recombinant human hyaluronidase (Hylenex) 900 IU with betamethasone 8 mg in a total volume of 5 mL, injected through the same stopcock at the target level. The smaller volume reflects the different intent: the enzyme acts locally on the hyaluronan matrix of the scar, and a large dilution would disperse it away from the tissue it is meant to soften.

#### 4.4.7. Indwelling Catheter, Second-Day Administration and Inpatient Observation

The catheter was not removed at the end of the procedure. It was secured to the skin with a sterile dressing and left in place for the first night, so that a second administration could be given the following day without an additional puncture. On the following day, at the bedside, a top-up bolus of 5 mL was injected through the indwelling catheter, consisting of levobupivacaine 2.5 mg/mL, 2 mL; methylprednisolone acetate 20 mg (0.5 mL); and normal saline 2.5 mL (final volume 5 mL, levobupivacaine 1 mg/mL), all of which was administered. The catheter was then withdrawn under gentle, steady traction and the entry site dressed. Immediately after removal, the catheter was inspected along its whole length and the tip examined to confirm that it was intact and that no fragment had been retained—a check performed and documented in every case, since retention of a sheared fragment is the principal device-related complication of catheter adhesiolysis and is far easier to exclude at the bedside than to diagnose later on imaging.

The purpose of the second administration is pharmacological rather than mechanical, and its rationale is theoretical: no comparative study has tested whether the second administration adds benefit over a single-session procedure, and it should be read as a protocol rationale rather than an established therapeutic advantage. The adhesiolysis performed on day one restores access to a nerve root that was previously shielded by scar, but the anti-inflammatory effect of a single corticosteroid dose is delivered into a space that is still acutely irritated by the procedure itself. Repeating the injection at 24 h is intended to exploit the window of enhanced diffusion created by the lysis, to prolong the interruption of nociceptive input during the period of maximal post-procedural inflammation, and to do so at a fraction of the risk and cost of a second percutaneous access. The catheter is removed immediately afterward, limiting the duration of indwelling access to a single night.

Patients were admitted for 48 h and remained in hospital for two nights. The procedure was performed on day one and the catheter left in situ for the first night; the top-up injection and catheter removal took place on day two, after which patients were observed for a further night and discharged on day three. The second night of observation is not required by the catheter, which has already been removed, but by the peri-procedural risk profile: delayed complications of an epidural intervention—expanding hematoma, evolving neurological deficit, early infection or a post-dural-puncture headache—characteristically declare themselves after the first 24 h rather than immediately, and a patient who has received a long-acting local anesthetic may not report a developing deficit reliably until the block has fully regressed.

Antibiotic cover was continued while the catheter remained in situ: a second dose of cefazolin 1 g intravenously was given on the morning of day two, before the top-up injection and catheter removal, with clarithromycin again substituted in beta-lactam-allergic patients. No antibiotic was administered after the catheter had been withdrawn.

The entry site was inspected before each injection and again at removal, and patients were monitored throughout for local or systemic signs of infection, for new or progressive neurological symptoms, for motor block regression, and for catheter displacement.

### 4.5. Indications, Safety and Clinical Limitations

#### 4.5.1. Matching the Technique to the Persistent Spinal Pain Syndrome Type

As noted in the Introduction, the persistent spinal pain syndrome taxonomy [11,12] has a technical consequence that is easy to overlook. The two types differ in the state of the epidural space itself, and therefore in which component of the procedure is doing the work.

Where there has been no operation, the space is usually patent; the catheter can be navigated, the obstruction is often a filmy band or a narrowed foramen, and much of the benefit derives from delivering drug where it could not previously go. Where there has been surgery, the space may be densely and circumferentially scarred; a catheter tip can create a channel through such tissue but cannot dissolve it, and the enzymatic component becomes correspondingly more important. Framing the choice this way turns a taxonomic distinction into an intra-procedural decision guide, and that is how it is used in the intra-procedural decision algorithm presented in Section 4.4.3—with the caveat, developed below, that this use has not been validated.

The distinction between the two PSPS types may help the operator anticipate which component of the problem is likely to dominate, although this use of the taxonomy for procedural selection has not been validated in comparative studies and is presented here as a working framework rather than an established algorithm. In type 1, where there has been no previous surgery, the epidural space is usually accessible, and the difficulty is compression combined with chemical sensitization of the root. Mechanical lysis with targeted drug delivery is expected to be sufficient more often; the enzymatic component is expected to add less, and the case for adding neuromodulation rests on the neuropathic features rather than on the scar. In type 2, where surgery has produced dense and often circumferential fibrosis, mechanical lysis alone frequently fails to restore diffusion. This is where the enzymatic component is most likely to be useful, and where a balloon catheter or epiduroscopy may be needed if the catheter cannot reach or cannot open the target.

Framing the choice this way also clarifies what our clinical series [13] can and cannot show. Its three options were not randomly assigned but selected according to pathology and intra-procedural findings, which is exactly the reasoning set out above. The resulting groups therefore differ in disease as well as in treatment, and comparisons between them are descriptive rather than causal.

#### 4.5.2. Where the Procedure Fits in the Treatment Pathway

The place of these procedures in the treatment sequence deserves comment. Van Boxem et al. [32] place pulsed radiofrequency adjacent to the ganglion after the level has been confirmed by a diagnostic block, and position epiduroscopy and adhesiolysis at the end of the sequence—after pulsed radiofrequency has proved insufficient, alongside spinal cord stimulation, and with the caveat that they be performed in a study context in specialized centers.

The configuration described here inverts that sequence, and this inversion is a proposal based on anatomical reasoning and on single-center experience, not a position established by comparative studies. It does not wait for one to fail before attempting the other: both are performed at the same target within a single procedure, and a second pharmacological administration follows through the indwelling catheter the next day. The justification is mechanistic. The ganglion that pulsed radiofrequency treats sits in an environment that helps to keep it sensitized—scarred, poorly perfused, impermeable to injected drug—and adhesiolysis alters precisely that environment while restoring diffusion at the same site. Separating them by months treats two aspects of one problem as though they were two problems. Observations from our cohort (unpublished data, under review [13])—in particular a greater improvement in disability where pulsed radiofrequency was added—are consistent with that reasoning without establishing it. In practical terms, the technique may be considered as an interventional option in selected patients—those with a concordant dermatomal pain, a neuropathic component, imaging showing a plausible target and an epidurogram showing impaired spread at that level—before surgical escalation in PSPS type 1, or when revision surgery is being considered in PSPS type 2; whether it should precede, follow or replace the alternatives in each group is a question that only comparative studies can answer.

#### 4.5.3. Complications, Pitfalls and How to Avoid Them

The reported complications of catheter-based adhesiolysis fall into a small number of categories, and their frequency, where it has been reported, is summarized in Table 3 together with the measure intended to reduce each risk. Most are made less likely, though not impossible, by adherence to the sequence described above. Pain at the puncture site and transient exacerbation of the existing pain are common and self-limiting. The serious complications are dural puncture with intrathecal injection, subdural spread, intravascular injection, epidural hematoma, infection ranging from soft-tissue abscess to epidural abscess and meningitis, direct trauma to a nerve root, and shearing or retention of the catheter.

Three checkpoints reduce the risk of most of these. Keeping the needle tip caudal to the S3 foramen on the antero-posterior projection keeps it below the termination of the dural sac. Aspirating for blood and for cerebrospinal fluid and then observing the response to a lidocaine test dose helps to identify intrathecal and intravascular placement before any therapeutic agent is given; it is worth remembering that a subdural block imitates a subarachnoid block but declares itself later, characteristically after 16 to 18 min, so a wait of at least twenty minutes is required before any injection whose intrathecal spread would be hazardous. Using an atraumatic, non-cutting needle and a catheter designed for the purpose, and never withdrawing the catheter through the needle, prevents shearing; inspecting the catheter along its whole length at removal confirms that no fragment has been retained.

Two further points are specific to this combination. Hyaluronidase increases the permeability of the interstitium, so it also accelerates the spread of anything misplaced; it must therefore follow, never precede, the safety checkpoints. And ionic contrast media must not be used, because inadvertent subarachnoid injection of an ionic agent has been associated with severe neurological complications including seizures.

The relationship between operator experience and the rate of serious complications is consistent across series, and the technique should be regarded as one requiring specific training rather than one that generalizes automatically from experience with simple epidural injections [15,46,47].

The incidence figures in Table 3 come almost entirely from a single retrospective series of the classical Racz technique with hypertonic saline [24], and from a two-center series in which adverse events were recorded within six months [25]; neither used the catheter described here, and no multicenter safety data exist for the combined protocol. The absence of major adverse events in our own 155 patients (unpublished data, under review [13]) is reassuring but is based on record review in a single center and cannot be generalized.

#### 4.5.4. What the Evidence Does Not Establish, and Who Might Be Selected

Four gaps are worth naming explicitly, and they qualify every mechanistic argument and every clinical suggestion made in this article. First, no study has isolated the contribution of the enzymatic component within a modern protocol that omits hypertonic saline, so the incremental value of hyaluronidase in this configuration is unknown. Second, no trial has compared the catheter-based techniques with each other; the choice between a steerable tip, a balloon, and an endoscope rests on mechanistic reasoning and on operator preference rather than on comparative data. Third, almost all series report beyond six months only sporadically, and estimates of durability are correspondingly weak. Fourth, function has usually been a secondary endpoint, and no adequately powered trial has tested disability as the primary outcome—which, given that the two domains may diverge, is a substantive omission rather than a technical one.

Until those gaps are addressed, patient selection rests on clinical and anatomical reasoning. The characteristics that in our experience and in the predictor studies [21,22] favor a good response are radicular pain with a clear dermatomal distribution and a neuropathic component, imaging that shows a plausible target at the corresponding level, a previous response of any duration to an epidural steroid injection, and an epidurogram that shows impaired spread concordant with the symptomatic level; the characteristics that predict a poorer response are repeated previous surgery, spondylolisthesis, severe foraminal stenosis and marked psychological distress. These are selection criteria for a procedure whose feasibility and rationale are well described; they are not evidence that selected patients do better with the combined protocol than with simpler alternatives.

## 5. Conclusions

Mechanical adhesiolysis, enzymatic lysis and pulsed radiofrequency of the dorsal root ganglion are proposed to act on different components of the same clinical problem: structural scarring and impaired drug diffusion, the ground substance of the scar itself, and the sensitized ganglion. A multifunctional steerable catheter makes it feasible to deliver all three at one anatomical target within a single procedure, with the epidurogram providing both the diagnostic target and the endpoint that documents whether the previously obstructed segment has been reopened. The technical description given here is intended to allow the procedure to be reproduced in another center; whether it is reproduced with the same results, and with the same safety, is a question that single-center experience cannot answer.

The feasibility and the rationale of the combined procedure should be distinguished from the evidence of clinical benefit. The components are each supported by literature of differing quality, the evidence for adhesiolysis itself is contested across specialties, and the combination has been tested in one randomized trial of 41 patients. The suggestion that the technique may be considered in selected patients—before surgical escalation in persistent spinal pain syndrome type 1, or when revision surgery is being considered in type 2—is a clinical proposal based on anatomical reasoning and on the experience reported, not a recommendation derived from studies that compared these treatment sequences. Selection currently rests on concordant dermatomal pain with a neuropathic component, a plausible target on imaging, a previous response to epidural steroid, and an epidurogram showing impaired spread at that level. Comparative studies that isolate the contribution of each component, that compare the catheter-based techniques with each other, and that measure function as a primary outcome over more than one year are needed before the place of the technique in treatment can be defined.

## Figures and Tables

**Table 1:** Principal clinical studies of catheter-based adhesiolysis, of hyaluronidase as an adjunct, and of dorsal root ganglion pulsed radiofrequency identified in the review.

Study	Design	*n*	Intervention	Follow-Up	Principal Finding
Vigneri 2020 [10]	RCT, double-blind	41	DRG-PRF + adhesiolysis vs. adhesiolysis alone	6 months	NRS −3.43 vs. −1.75 at 1 month; 48% vs. 10% responders at 6 months
Manchikanti 2012 [16]	RCT	120	Adhesiolysis vs. caudal epidural injection	2 years	82% vs. 5% with ≥50% relief and functional gain; mean 6.4 procedures
Yousef 2010 [17]	RCT, double-blind	38	Caudal steroid + hypertonic saline ± hyaluronidase	12 months	Early relief in both arms; sustained relief only with hyaluronidase
Kose 2023 [21]	Retrospective cohort	193	Adhesiolysis after failed epidural steroid injection	12 months	56.2% with ≥50% relief; depression, spondylolisthesis, previous surgery and severe foraminal stenosis predicted failure
Lee 2013 [22]	Retrospective cohort	65	Adhesiolysis in stenosis refractory to transforaminal injection	3 months	54% with ≥50% relief of back pain; same predictors of failure
Choi 2014 [23]	Case series	4	Balloon-inflatable catheter after failed adhesiolysis	>7 months	Relief and longer walking distance where a conventional catheter had failed
Helm 2016 [18]	Systematic review	—	Percutaneous and endoscopic adhesiolysis	—	Effective and safe in trained hands; strongest effect at 3–6 months
Brito-García 2019 [19]	Systematic review	—	Epidural adhesiolysis for failed back surgery syndrome	—	No RCT on efficacy; two RCTs on effectiveness with serious methodological flaws; evidence on effectiveness and safety judged insufficient
Helm 2020 [20]	Commentary	—	Reply to the 2019 systematic review	—	Argues that the review misclassified the trials; maintains that adhesiolysis is effective and safe
Talu 2003 [24]	Retrospective series	250	Epidural neuroplasty (Racz technique)	—	Dural puncture 4.4%, catheter shearing 1.2%, epidural abscess 1.2%, meningitis 0.8%
Choi 2017 [25]	Retrospective, two centers	543	Percutaneous adhesiolysis with 5% vs. 10% hypertonic saline	6 months	Similar pain relief; transient adverse events in 3 patients (0.6%)
Racz 2008 [26]	Narrative review	—	Percutaneous lysis of epidural adhesions	—	Supports safety and efficacy; protocols highly heterogeneous

**Table 2:** Comparison of the principal catheter-based and endoscopic adhesiolysis techniques.

	Classical Racz	Steerable Multifunctional Catheter	Balloon-Inflatable Catheter	Epiduroscopy
Mechanism of lysis	linear, tip advancement	linear, steerable tip	radial dilation	direct vision, instrumented
Visualization	epidurography	epidurography	epidurography	direct endoscopic
Enzymatic component	hyaluronidase 1500 IU	hyaluronidase 900 IU (recombinant)	optional	optional
Hypertonic saline	classically yes	no	no	no
Neuromodulation	no	yes—bipolar DRG-PRF at the tip	no	no
Protocol	1–3 days	2 days	single session	single session
Best suited to	PSPS type 2, dense scar	either type; neuropathic features	foraminal or central narrowing	diagnostic uncertainty, repeated failure
Main limitations	hypertonic saline risk, multi-day	cost of the device	cost, limited evidence base	equipment, learning curve, irrigation volume

**Table 3:** Adverse events reported after catheter-based epidural adhesiolysis and dorsal root ganglion pulsed radiofrequency, their reported frequency, and the measures used in the present protocol to reduce each risk.

Adverse Event	Reported Frequency (Source)	Risk-Reduction Measure in the Present Protocol
Dural puncture, intrathecal spread	11/250 (4.4%) in a series of 250 neuroplasties [24]; none in the 155 patients of our cohort (unpublished data [13])	Needle tip kept caudal to the S3 foramen; aspiration; lidocaine test dose with neurological check; contrast under live fluoroscopy
Subdural or intradural catheter migration	3/250 (1.2%) [24]	Test dose followed by an observation interval of at least 20 min, since subdural block declares itself after 16–18 min; repeat contrast before every therapeutic injection
Intravascular placement	Blood aspiration 39/250 (15.6%); catheter migration into an epidural vein 8/250 (3.2%) [24]	Aspiration before every injection; vascular uptake of contrast sought on live fluoroscopy; impedance outside 200–500 Ω prompts repositioning
Catheter shearing, retained fragment	3/250 (1.2%) [24]	Catheter designed for the purpose, never withdrawn through the needle; inspected along its whole length at removal
Transient numbness or paresthesia; transient exacerbation of pain	25/250 (10%) [24]; common and self-limiting in most series [18]	Low anesthetic concentration (1.25 mg/mL), fractionated injection; sensory and motor stimulation testing before pulsed radiofrequency
Hypotension during injection	12/250 (4.8%) [24]	Continuous hemodynamic monitoring; fractionated injection
Infection: entry site, epidural abscess, meningitis	Entry site 8/250 (3.2%); epidural abscess 3/250 (1.2%); meningitis 2/250 (0.8%) [24]; none in our cohort	Antibiotic prophylaxis before puncture and while the catheter is in situ; indwelling access limited to one night; entry site inspected before each injection and at removal
Epidural hematoma	Rare; case reports [46,47]	Coagulation screening and anticoagulant management before the procedure; inpatient observation for 48 h
New or persistent neurological deficit	Rare; case reports [46,47]	Motor stimulation at 2 Hz before energy delivery; no energy if segmental contraction is elicited; temperature ceiling of 42 °C
Complications specific to pulsed radiofrequency	No lesion-related deficit reported in reviews of DRG-PRF [32,33]; transient post-procedural pain is common	Impedance check; sensory threshold ≤ 0.5 V at 50 Hz; automatic voltage titration at the 42 °C ceiling
Any transient adverse event	3/543 (0.6%) in a modern two-center series [25]; 19 events among 110 patients in the observational studies pooled by a 2019 systematic review [19]	—

## Data Availability

The anonymized dataset is available from the corresponding author on reasonable request. This article is a narrative review and technical report and does not present original clinical outcome data; the clinical cohort from which the illustrations and the summary figures in Section 4.2 derive is the subject of a separate manuscript, currently under review [13], in which the full methods, results and the completed STROBE checklist are provided, and the figures quoted here are unpublished data presented only in summary form.

## Supplementary Materials

The following supporting information can be downloaded at: https://www.mdpi.com/article/10.3390/jcm15208094/s1. Supplementary Material S1: Table S1, MEDLINE (PubMed) search strings, run on 14 September 2026, inception to 31 December 2025.

## Abbreviations

Abbreviation	Term
DN4	Douleur Neuropathique 4 questionnaire
DRG-PRF	Pulsed radiofrequency of the dorsal root ganglion
IL-1β	Interleukin-1 beta
IL-6	Interleukin-6
NMDA	N-methyl-D-aspartate
NRS	Numerical rating scale
PLA2	Phospholipase A2
PSPS	Persistent spinal pain syndrome
STROBE	Strengthening the Reporting of Observational Studies in Epidemiology
TNF-α	Tumor necrosis factor alpha
