# Supplementary material for: Epidural Adhesiolysis and Dorsal Root Ganglion Pulsed Radiofrequency Through a Single Multifunctional Catheter: A Narrative Review and Illustrated Technical Report
Source: J Clin Med. 2026 Oct 19;15(20):8094. doi: 10.3390/jcm15208094 (PMC13705801; doi:10.3390/jcm15208094)
Supplement: Supplementary file 1 [file jcm-15-08094-s001.zip › jcm-4595525-supplementary.pdf]

Supplementary Material S1

Epidural Adhesiolysis and Dorsal Root Ganglion Pulsed Radiofrequency Through a Single Multifunctional Catheter: A Narrative Review and Illustrated Technical Report

S1.1 Literature search strategy

This article is a narrative review. The search described here was designed to orient the reader within the literature on catheter-based epidural adhesiolysis and dorsal root ganglion pulsed radiofrequency, not to identify every eligible study, and no protocol was registered. MEDLINE (via PubMed) and Embase were searched from database inception to 31 December 2025, without language restriction. To make the search reproducible, the MEDLINE search was re-run on 14 September 2026 with the exact strings reported in Table S1, and the record counts in Figure S1 refer to that run.

MEDLINE and Embase were chosen because they are the two largest bibliographic databases of biomedical literature and have complementary coverage: MEDLINE indexes the core clinical and neuroscience journals, whereas Embase provides broader coverage of European journals, of drug and device literature and of conference abstracts, which is relevant to a technique whose development took place largely in Europe and whose device-specific evidence is sparse. The Cochrane Library was not searched separately because its trial register is derived from MEDLINE and Embase, and grey-literature sources were not searched because the review is not a systematic synthesis.

Four search blocks were built, one for each component of the technique, combining Medical Subject Headings (MeSH) with free-text terms in title and abstract. Results were exported, duplicates across blocks were removed, and records were screened on title and abstract by a single reviewer (G.L.B.) for relevance to the scope of the review. Reference lists of the retrieved articles, the guideline documents of the American Society of Interventional Pain Physicians, and the authors' previous work were hand-searched. Priority was given to randomized trials, systematic reviews and guideline documents; study selection was not duplicated and reasons for non-retention were not recorded individually.

Table S1. MEDLINE (PubMed) search strings, run on 14 September 2026, inception to 31 December 2025.

Block	Concept	Search string (PubMed syntax)	Records
1	Epidural adhesiolysis	("epidural adhesiolysis"[Title/Abstract] OR epidurolysis[Title/Abstract] OR "lysis of adhesions"[Title/Abstract] OR "epidural neuroplasty"[Title/Abstract] OR Racz[Title/Abstract]) AND (epidural[Title/Abstract] OR "Epidural Space"[MeSH Terms])	197
2	Dorsal root ganglion pulsed radiofrequency	("Pulsed Radiofrequency Treatment"[MeSH Terms] OR "pulsed radiofrequency"[Title/Abstract]) AND ("Ganglia, Spinal"[MeSH Terms] OR "dorsal root ganglion"[Title/Abstract]) AND (lumbar[Title/Abstract] OR lumbosacral[Title/Abstract] OR radicular[Title/Abstract] OR "Radiculopathy"[MeSH Terms])	115
3	Hyaluronidase, epidural	("Hyaluronoglucosaminidase"[MeSH Terms] OR hyaluronidase[Title/Abstract]) AND (epidural[Title/Abstract] OR "Injections, Epidural"[MeSH Terms] OR "failed back surgery"[Title/Abstract] OR "persistent spinal pain"[Title/Abstract])	70
4	Epiduroscopy and balloon catheters	(epiduroscopy[Title/Abstract] OR "spinal endoscopy"[Title/Abstract] OR "epidural endoscopy"[Title/Abstract] OR balloon[Title/Abstract]) AND (adhesiolysis[Title/Abstract] OR "epidural fibrosis"[Title/Abstract] OR "failed back surgery"[Title/Abstract] OR "Failed Back Surgery Syndrome"[MeSH Terms])	130
		Total before deduplication / unique records	512 / 442

MeSH terms used: Epidural Space; Pulsed Radiofrequency Treatment; Ganglia, Spinal; Radiculopathy; Hyaluronoglucosaminidase; Injections, Epidural; Failed Back Surgery Syndrome. Embase was searched at the time of writing with the corresponding Emtree terms (epidural space; pulsed radiofrequency; spinal ganglion; radiculopathy; hyaluronidase; epidural drug administration; failed back surgery syndrome) and

the same free-text terms; the Embase record counts were not archived and the Embase search was not re-run for this supplement.

The record counts obtained from this search, and the provenance of the 46 references cited in the article (13 retrieved by the database search, 31 identified from reference lists, the authors' previous work, co-authors' suggestions and the guideline, taxonomy and background literature, and 2 not indexed in MEDLINE), are presented in Figure 1 and Section 3 of the main text. The counts refer to the MEDLINE/PubMed search re-run on 14 September 2026 with a cutoff of 31 December 2025; Embase was searched with the same terms but its record counts were not archived. Study selection was performed by a single reviewer and was not duplicated, and no risk-of-bias assessment was performed.
